# Presenting the ***Compendium Isotoporum Medii Aevi***, a Multi-Isotope Database for Medieval Europe

**DOI:** 10.1038/s41597-022-01462-8

**Published:** 2022-06-21

**Authors:** Carlo Cocozza, Enrico Cirelli, Marcus Groß, Wolf-Rüdiger Teegen, Ricardo Fernandes

**Affiliations:** 1 Institut für Vor- und Frühgeschichtliche Archäologie und Provinzialrömische Archäologie, and ArchaeoBioCenter, Ludwig-Maximilians-Universität München, Geschwister-Scholl-Platz 1, 80539 München, Germany; 2 Department of Archaeology, Max Planck Institute for the Science of Human History, Kahlaische Str. 10, 07745 Jena, Germany; 3 Dipartimento di Scienze e Tecnologie Ambientali Biologiche e Farmaceutiche (DiSTABiF), and Mediterranean bioArchaeological Research Advances (MAReA) centre, Università degli studi della Campania “Luigi Vanvitelli”, Via Vivaldi 43, 81100 Caserta, Italy; 4 Dipartimento di Storia Culture Civiltà, Alma Mater Studiorum Università degli Studi di Bologna, Piazza San Giovanni in Monte 2, 40124 Bologna, Italy; 5 School of Archaeology, University of Oxford, 1 Parks Road, OX1 3TG Oxford, UK; 6 Arne Faculty of Arts, Masaryk University, Nováka 1, 602 00 Brno, Czech Republic

**Keywords:** Archaeology, Stable isotope analysis

## Abstract

Here we present the *Compendium Isotoporum Medii Aevi (CIMA)*, an open-access database gathering more than 50,000 isotopic measurements for bioarchaeological samples located within Europe and its margins, and dating between 500 and 1500 CE. This multi-isotope (δ^13^C, δ^15^N, δ^34^S, δ^18^O, and ^87^Sr/^86^Sr) archive of measurements on human, animal, and plant archaeological remains also includes a variety of supporting information that offer, for instance, a taxonomic characterization of the samples, their location, and chronology, in addition to data on social, religious, and political contexts. Such a dataset can be used to identify data gaps for future research and to address multiple research questions, including those related with studies on medieval human lifeways (i.e. human subsistence, spatial mobility), characterization of paleo-environmental and -climatic conditions, and on plant and animal agricultural management practices. Brief examples of such applications are given here and we also discuss how the integration of large volumes of isotopic data with other types of archaeological and historical data can improve our knowledge of medieval Europe.

## Abstract (summary)

**Table:** 

Measurement(s)	Isotopes
Technology Type(s)	Mass Spectrometry
Factor Type(s)	Carbon isotopes • nitrogen isotopes • sulfur isotopes • oxygen isotopes • strontium isotopes
Sample Characteristic - Organism	Humans • Animals • Plants
Sample Characteristic - Location	Europe and Periphery

## Background & Summary

The Middle Ages (c. 500 to 1500 CE) is a formative period of European history. It was marked by major transformations in political and economic systems, vast population movements, violent armed conflicts, climate change, development of religious movements, and technological innovations, albeit with regional variations^1–9^. The study of such historical phenomena has been predominantly based on written sources although these may vary in quality and representativity^10^. In particular, the lifestyles of lower socioeconomic classes are often mis- or under-represented given their illiteracy. Knowledge gaps can be reduced by isotopic analyses of human remains from which it becomes possible to build iso-biographies describing the diets and spatial mobility of single individuals from across socioeconomic, religious, and cultural spectra^11–29^. Isotopic analyses of animal and plant remains have also been employed in medieval contexts to reconstruct past climatic and environmental conditions plus to investigate economic and agricultural activities^30–43^.

In the late 1970’s, stable carbon isotope analysis of human remains was first employed for paleo-diet reconstruction^44,45^. Since then, the use of isotopic methods in archaeological research has expanded following several developments in isotope ratio mass spectrometry methods and lab pretreatment protocols that increased the number of measurable isotopic ratios across a wide variety of materials^46–48^. Such developments have allowed for a larger number of applications in archaeological research and for more accurate and precise assessments of past phenomena. The reconstruction of past human subsistence, nutrition and spatial mobility, the study of past animal and crop management practices, or the reconstruction of paleo-environments and -climates are just some examples that illustrate the importance of isotopic methods in archaeological research^49–57^. This is also evident from the exponential growth in recent decades in the number of archaeological publications reporting isotopic results^58^. Once collected and curated, amassed isotopic data can be subject to meta-analyses from which it is possible to investigate past human and natural phenomena at varying spatial and temporal scales^59–61^.

Recent databases have partially compiled isotopic data for the European medieval world^62,63^. Here we present the open-access CIMA (*Compendium Isotoporum Medii Aevi*) database, the first isotopic database to comprise the full extent of the medieval period across the entirety of Europe and its margins. This database also includes for the first time all types of bioarchaeological remains (plants, animals, and humans) and isotopic measurements (δ^13^C, δ^15^N, δ^34^S, δ^18^O, and ^87^Sr/^86^Sr) on bulk organic remains and on tooth increments. To address various historical questions, CIMA includes metadata that characterizes the political, religious, and social context of listed samples. Here we describe CIMA and briefly illustrate its research potential.

## Methods

The collection of published isotopic measurements for medieval Europe and its margins began in November 2019 and was completed in May 2020. Since then, regular updates have been made to the database following the publication of new data. Isotopic measurements were obtained from journal articles, book chapters, archaeological reports, and academic dissertations available in different languages (Portuguese, Italian, English, Spanish, French, Swedish, Dutch, and German). Publications were located through a web search using scientific search engines (e.g. Google Scholar) employing different combinations of keywords such as “Medieval”, “Isotope” plus geographical or cultural tags (e.g. “Italy” or “Longobard”). We also relied on thorough readings of publications’ bibliography through which several additional isotopic datasets were located.

Data collection was mostly limited to Europe for samples dated between c. 500 and 1500 CE. However, isotopic measurements from non-European regions presenting cultural or religious affinities with medieval Europe (e.g. Norse populations in Greenland or Christian Crusaders in Jordan or Palestine) were also added. In this compilation, we included isotopic measurements (δ^13^C, δ^15^N, δ^34^S, δ^18^O, and ^87^Sr/^86^Sr) of human and animal bone and tooth collagen (including tooth increments), bone bioapatite and tooth enamel, and plant organic remains. We did not include single compound measurements but this is planned for future CIMA updates once more data becomes available.

The CIMA database includes meta-data on the historical, cultural, religious, and social context of the samples. This information was collected both from primary and secondary publications on sites and individuals. Each isotopic measurement has an internal ID (sequential integer assignment) together with original IDs, as per primary sources, on each individual sample plus also, when available, for archaeological context and site. In some instances, isotopic values were only reported as a population mean. Whenever possible we contacted publication authors to obtain individual measurements plus additional contextual information. If this was not possible, data entries were flagged (data fields list the number of measurements included in the mean calculation).

A detailed description of the database metadata structure is given in Supplementary Information File S1. To maintain data consistency, we had at times to perform data conversions. Examples of this are reported human osteological descriptions (e.g. osteologically determined ages are listed in the database using the Buikstra and Ubelaker system^64^). The assignment of political, cultural, social and religious values is based on the archaeological, historical, and chronological context as reported by academic publications. This often does not assign a specific individual to a certain religious or political group but rather places a burial population within a site or even regional context. In cases where such an assignment is ambiguous we include in the database the various possibilities (e.g. religious assignment may be listed as “pagan; Christian”).

Each database entry is georeferenced using decimal coordinates (“Latitude”; “Longitude”) relative to the WGS84 system. Whenever available we used the geographical coordinates as reported in the original publication. If these were not available, the archaeological site was located and georeferenced using Google Earth. It was not always possible to locate the geographic center of archaeological sites. In such cases we identified the smallest administrative unit and used its geographical center. The field “unc. Radius (km)” gives an estimate on the radius of uncertainty (in km) for the location of a site.

The chronology of each sample is given as a temporal interval (“Min. Year (95%)”; “Max. Year (95%)” in years CE). Also included are data fields (“General Period(s)”; “Additional Chronological Tags”) that describe chronological categories as text strings. The temporal interval was assigned following a hierarchical approach. Whenever direct dating of samples was available this was used (e.g. from radiocarbon dating). Otherwise, and successively, we employed the dating of the burial context, burial site, and overall culture.

## Data Records

**Fig. 1:**
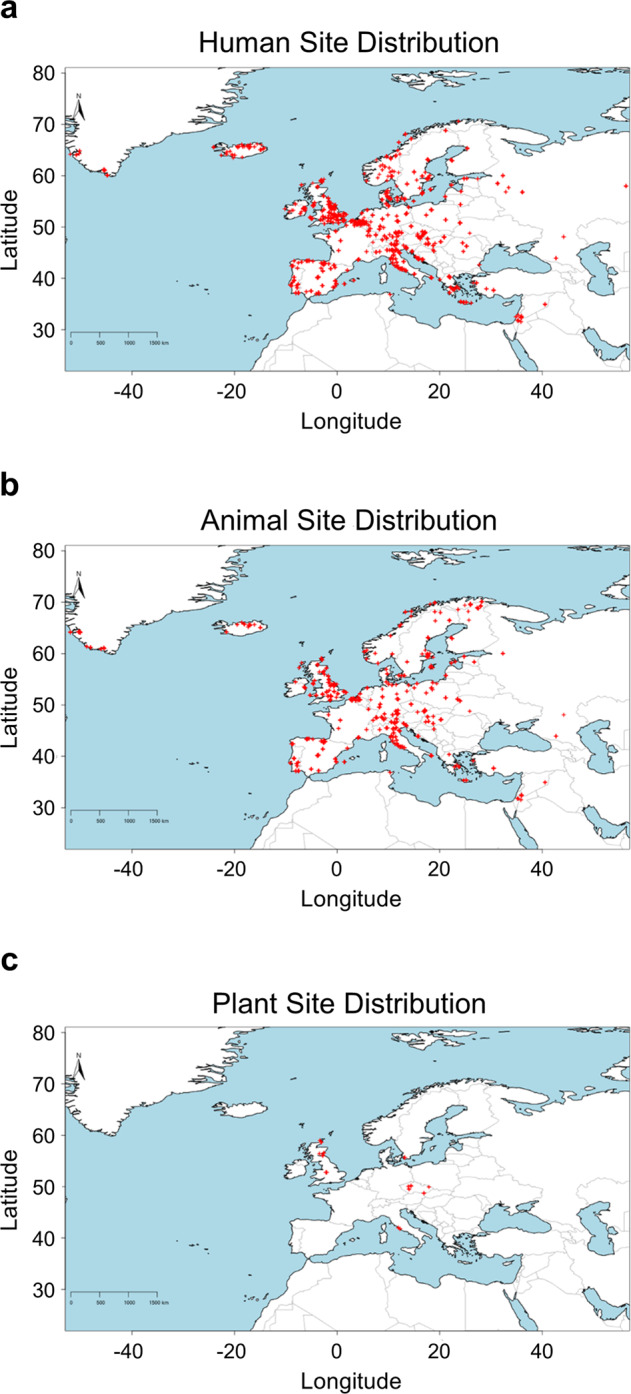
Spatial distribution of human (**a**), animal (**b**), and plant (**c**) site locations for data compiled within CIMA.

CIMA is organized into three separate datasets according to sample categories (humans, animals, plants) made available as Excel and CSV files. It consists of 17,756 human, 4946 animal, and 164 plant entries. Isotopic data was collected from 358 primary sources (full list given in Supplementary Information File S2). The total number of δ^13^C, δ^15^N, δ^34^S, δ^18^O, and ^87^Sr/^86^Sr measurements included in the database is 50,153. Most of the collected data originates from archaeological sites located in the UK (24.1%), followed by Italy (10.8%), Spain (9.6%), and Germany (8.0%). The spatial distribution of archaeological sites included in CIMA is shown in Fig. 1. This reveals a major data gap for France (3.1% of data) which is compounded by its size and importance in medieval European history. Additional summaries and descriptions of human, animal, and plant data can be found in Supplementary Information File S1 and S3.

The CIMA datasets^65^ (10.48493/s9nf-1q80) are made available via the Pandora data platform (https://pandora.earth/) within the MATILDA data community (https://pandoradata.earth/organization/matilda-a-repository-for-medieval-bioanthropological-databases) that collects historical and archaeological data relevant for the study of medieval Europe. Depending on assigned roles, MATILDA data community members may create/edit datasets and assign to these new DOIs. It is both possible to store datasets and to provide links to external compilations having previously assigned DOIs. Under this setup, individual researchers, research groups, museums, and laboratories can easily make available their medieval isotopic data as individual datasets within the MATILDA data community. This data is then incorporated into the CIMA master files following the predefined metadata standards. These master files include reference data fields that identify both primary sources with original data (“Reference”; “Link”; “DOI”; “Publication date”) plus data compilations (“Compilation Reference”; “Compilation Link”; “Compilation DOI”; “Compilation Publication Year”). Under this system it is possible to easily track and acknowledge both previous data production and data compilation efforts. CIMA and MATILDA are open to new memberships and data contributions from research groups and individuals performing isotopic research on medieval Europe.

Another feature made available via the Pandora platform is the possibility of creating data networks linking separate datasets. One such example is a network of isotopic datasets (https://pandoradata.earth/group/isomemo-group) which are part of the IsoMemo initiative (https://isomemo.com/). IsoMemo is a collaborative network of independent isotopic databases. It includes several archaeological isotopic databases allowing for comparative studies at various spatiotemporal scales^66–70^.

## Technical Validation

The database lists standard measures (“Collagen Yield”; “%C”; “%N”; “Atomic C:N ratio”; “Atomic C:S ratio”; “Atomic N:S ratio”) employed to assess collagen preservation and establish the reliability of isotopic measurements for dietary or mobility studies^71–74^. Measurements of preservation criteria falling outside of accepted ranges were kept in the database since these can be used in studies related to sample preservation. However, for dietary or mobility studies they should be filtered out prior to data analysis.

Carbon stable isotope ratios are typically measured using an isotope ratio mass spectrometer (IRMS). However, some publications report measurements made using accelerator mass spectrometry (AMS). These are usually produced during radiocarbon dating and employed to correct radiocarbon concentrations for isotopic fractionation that may take place during sample preparation (e.g. combustion, graphitization) and machine measurement. The AMS and IRMS δ^13^C values may differ considerably although this varies across laboratories and with sample preparation and measurement techniques^75,76^. In CIMA we employed separate fields to report IRMS (“IRMS δ^13^C Collagen”; “IRMS δ^13^C Collagen unc.”; “δ^13^C Carbonate”; “δ^13^C Carbonate unc.”) and AMS (“AMS δ^13^C Collagen”; “AMS δ^13^C Collagen unc.”) δ^13^C values. Uncertainty associated with isotopic measurements is marked in database fields using “unc.”.

Oxygen isotopic ratios are frequently measured on carbonates although phosphate measurements are at times reported. In addition, these measurements may also be reported relative to VPDB (Vienna Pee Dee Belemnite) or VSMOW (Vienna Standard Mean Ocean Water) standards. In some studies, for instance on spatial mobility, conversions are made to report δ^18^O measurements relative to the same standard and molecular ions by relying on previous experimental work^77–79^. In CIMA, δ^18^O results are listed using the standard and molecular ion as given in original publication (“δ^18^O Carbonate (VPDB)”; “δ^18^O Carbonate (VPDB) unc.”; “δ^18^O Carbonate (VSMOW)”; “δ^18^O Carbonate (VSMOW) unc.”; “δ^18^O Phosphate (VPDB)”; “δ^18^O Phosphate (VPDB) unc.”; “δ^18^O Phosphate (VSMOW)”; “δ^18^O Phosphate (VSMOW) unc.”). In addition, conversions may be made to calculate the δ^18^O of drinking water^77–79^. Some publications give only these values and are listed in CIMA using a separate field (“δ^18^O Drinking Water (if not reported differently)”).

## Usage Notes

The CIMA compilation of medieval isotopic data can be employed for multiple research goals including: 1) paleoclimatic and paleoenvironmental studies; 2) investigating past human agricultural management practices; 3) and in the reconstruction of different aspects of past human lifeways such as diet, nutrition, and spatial mobility. In the following section we provide brief examples that illustrate this research potential and how data from this collection can be combined with non-isotopic data from the medieval period. We implemented an R-based toolkit to access online data records using a Shiny app interface (https://isomemoapp.com/)^80,81^. In addition, the app includes interactive dashboards for data modeling employing previously published Bayesian and non-Bayesian methods^59–61,82,83^. Further details on modeling methods employed in examples below are given in Supplementary Information File S4 and S5.

Stable carbon and nitrogen isotopic values from archaeological animal remains are a palimpsest of information on agricultural management practices (e.g. irrigation, manuring) and of how local vegetation is influenced by environmental/climatic conditions (e.g. precipitation, canopy effects, altitude, soil chemistry, etc.)^54,56,57,84^. To illustrate spatiotemporal isotopic variability in bone collagen for terrestrial animals from Medieval Europe we employed broad temporal (time bin 500 to 1000 CE *versus* 1000 to 1500 CE) and spatial (Europe plus sub-selections for England, Iberia, and Italy) divisions. The comparison was made for δ^13^C and δ^15^N IRMS values from domesticated herbivores (cattle/ovicaprids) and omnivores (pigs/chickens).

**Fig. 2:**
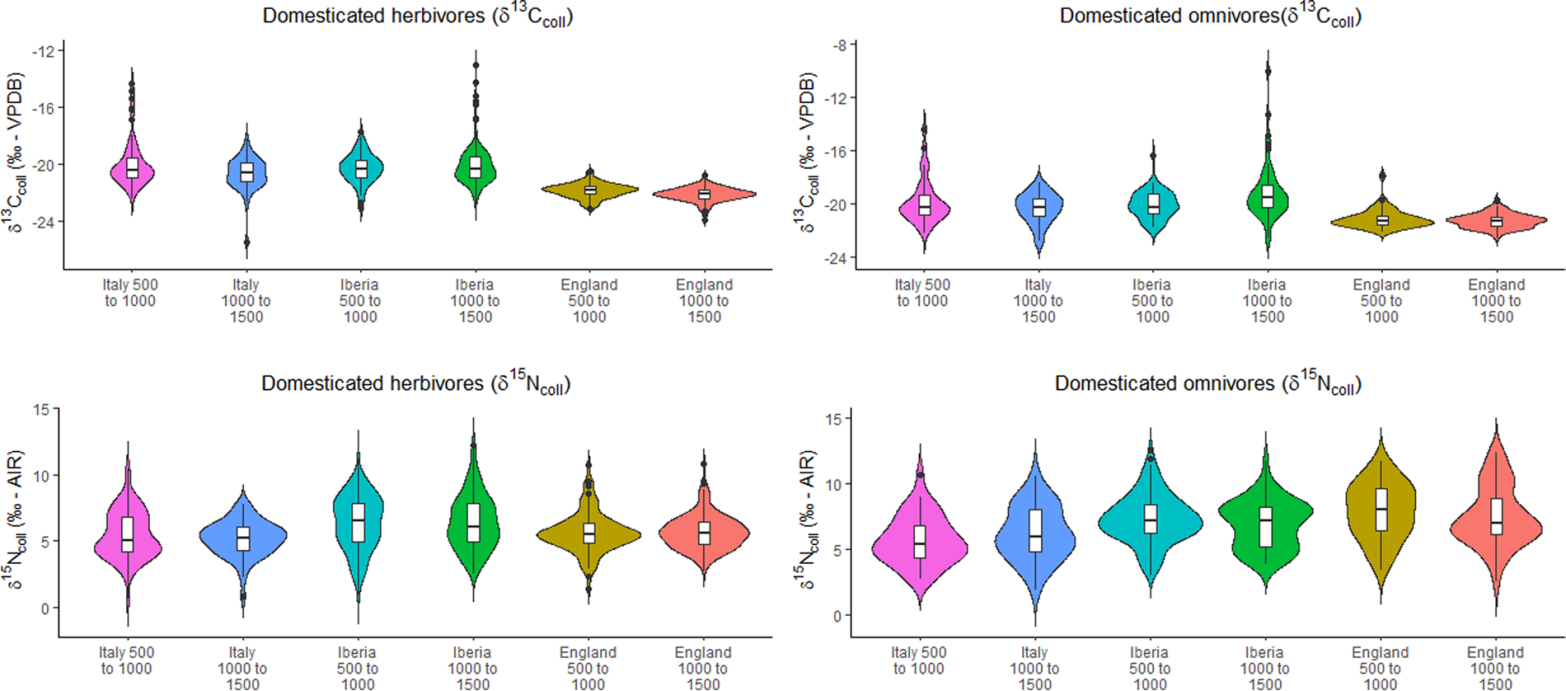
Violin plots showing a temporal comparison of δ^13^C and δ^15^N bone collagen values from domesticated herbivores and omnivores in Italy, Iberia, and England.

**Fig. 3:**
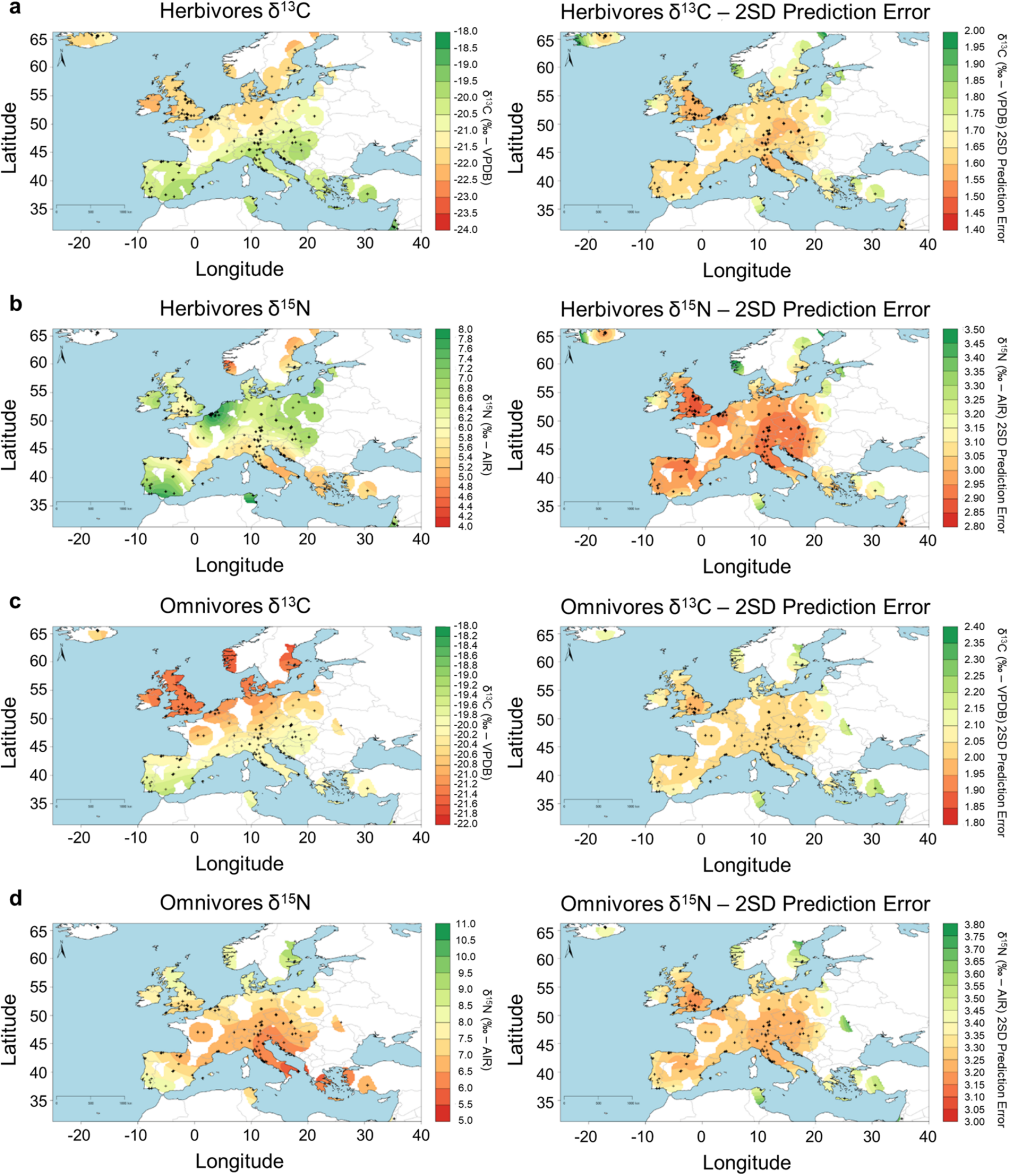
Spatial comparison of predicted δ^13^C and δ^15^N mean and associated errors (double the square root of the sum of the standard error plus the square of the population standard deviation) for domesticated animals. (**a**) δ^13^C herbivores; (**b**) δ^15^N herbivores; (**c**) δ^13^C omnivores; (**d**) δ^15^N omnivores.

The diachronic comparison using violin plots for selected regions (Fig. 2) and the observed spatial patterns for all combined periods (Fig. 3) show that Italy and Iberia have roughly similar distributions for both bone collagen δ^13^C and δ^15^N values and that these differ from England when it comes to domesticated herbivores. In the case of herbivore bone collagen δ^13^C, observed patterns likely reflect a higher water abundance and greater canopy effect in northern Europe although some of the more highly elevated δ^13^C values in southern Europe suggest animal consumption of C_4_ plants such as millet or sorghum or, in the case of Muslim Iberia, of sugarcane production wastes^11,84–86^. The δ^15^N values in England and Italy/Iberia are similar for omnivores but show narrower ranges for herbivores in England in spite of the considerably larger environmental variability in Iberia/Italy. Given that there are no visible temporal differences, this suggests that omnivores’ feeding and crop/vegetation management practices differ considerably within medieval England^87^.

The CIMA database allows for studies of the impacts that major historical developments had on different aspects of human lifeways such as diet. For instance, the collapse of the Western Roman Empire (476 CE), the splitting of its territories into separate kingdoms, and the subsequent territorial unification attempt during the Carolingian empire (800–887 CE) mark major historical transitions in Europe^6–8^. Different sources of historical and archaeological evidence point towards a higher diversification of farming and animal rearing in Late Roman to early medieval Europe, yet far from the intensive agricultural economy of the Roman Empire^85,88–91^. In concomitance, the arrival of migrating populations may have also modified local dietary practices^92^.

**Fig. 4:**
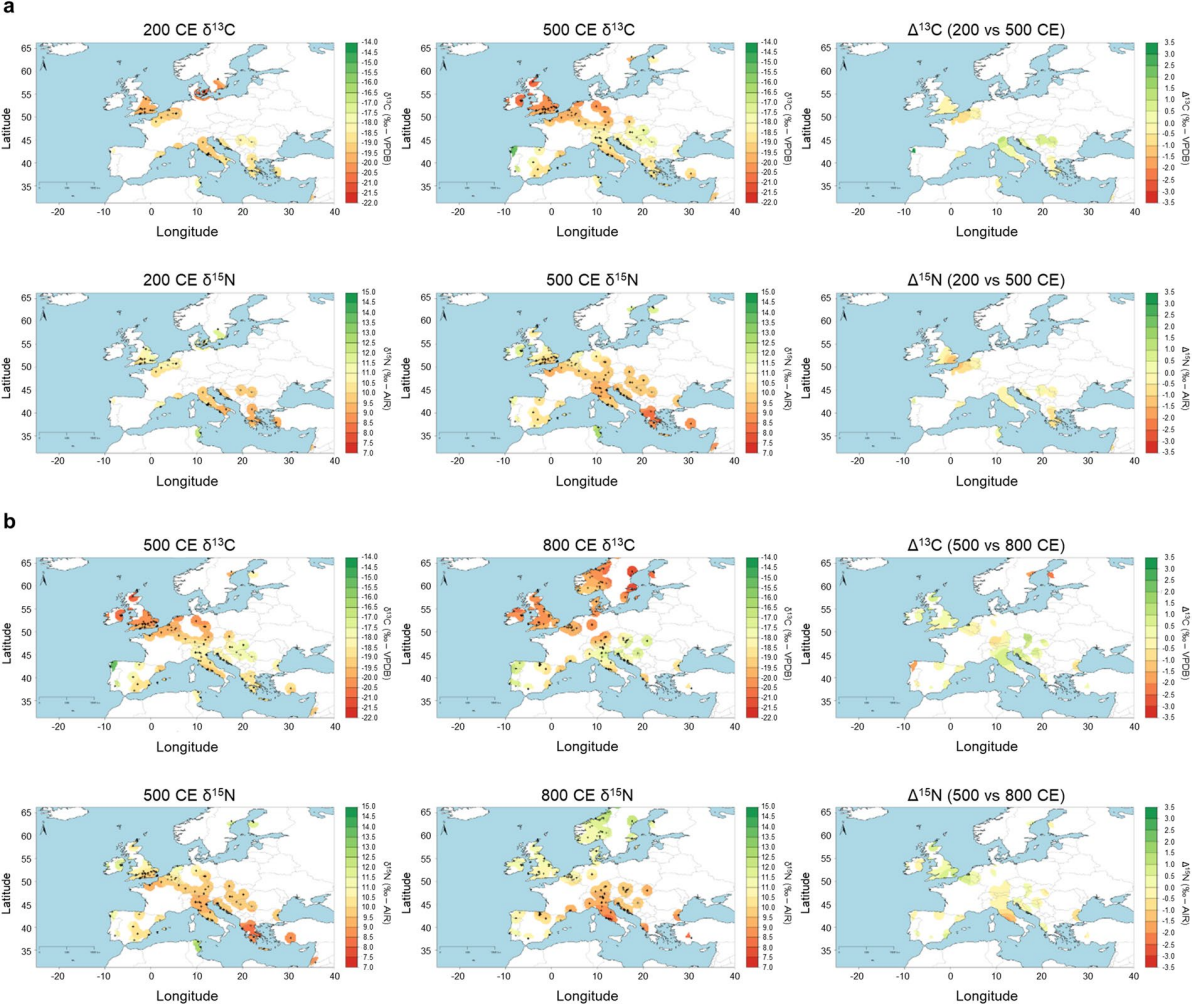
(**a**) Bayesian spatial estimates of δ^13^C and δ^15^N mean values for human bone collagen in 200 CE (left column) and 500 CE (middle column) and mapping of differences in isotopic values (Δ^13^C and Δ^15^N, right column). (**b**) Bayesian spatial estimates of δ^13^C and δ^15^N mean values for human bone collagen in 500 CE (left column) and 800 CE (middle column) and Bayesian mapping of differences in isotopic values (Δ^13^C and Δ^15^N, right column).

We combined CIMA medieval isotopic data with Roman isotopic data from the IsoArcH database^93^, to map and compare spatial distribution of human adult bone collagen carbon (δ^13^C - IRMS) and nitrogen (δ^15^N) stable isotopes for three time slices: 200 CE, 500 CE, and 800 CE (Fig. 4). This revealed regional differences in human isotopic values that reflect differences in diet and/or local isotopic baselines plus diachronic shifts associated with historical transitions. For instance, the comparison of the 200 and 500 CE time slices shows that in some regions (e.g. Galicia in northern Spain, northern Italy, and northern Balkans) there were increases in δ^13^C without major shifts in δ^15^N. This suggests larger consumption of C_4_ cereals (e.g. millet and/or sorghum) and/or products from animals foddered on these. Such a dietary shift may be the result of new incoming dietary traditions (e.g. Suebi in western Iberian^20^) but it should also be noted that the collapse of the Roman economic and agrarian systems reduced access to wheat and barley while millet and sorghum became commonly consumed by lower socioeconomic classes^85,86,94–96^. The comparison of the 500 CE and 800 CE time slices reveals regions with visible isotopic shifts. In northern Italy and the Balkans, the increase in δ^13^C values shows that the cultivation of C_4_ cereals increased through time^85,86,94–96^. In central Italy, there is a decrease in δ^15^N values. Here a reduction in animal sizes and a general shift towards silvopastoralism is consistent with a decline in the consumption of terrestrial animal protein and/or a decrease in animal δ^15^N values as consequence of free-roaming rearing practices^89,90^.

**Fig. 5:**
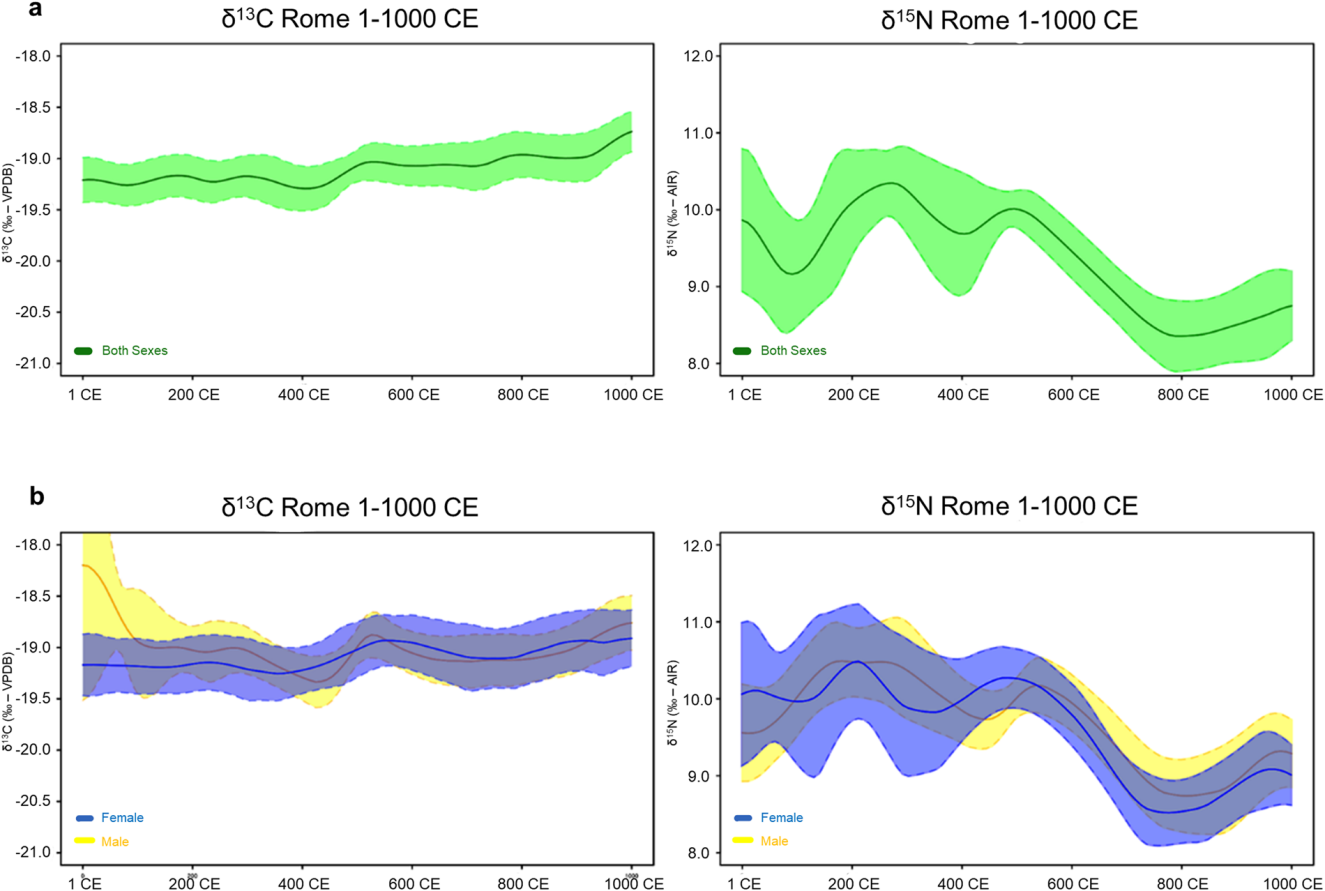
Temporal Bayesian plots for adult bone collagen δ^13^C and δ^15^N values for Rome. (**a**) both sexes δ^13^C and δ^15^N; (**b**) female *versus* male δ^13^C and δ^15^N.

Diachronic patterns in human lifeways may also be investigated for specific locations and offer insights into changes in medieval socioeconomic  structures. For instance, some historical sources suggest the existence of gender-based nutritional inequality in antiquity, although their extension beyond restricted communities (e.g. monastic) remains unknown^92,97,98^. Figure 5 shows temporal plots of adult human isotopic values classified according to osteological sex for the city of Rome between 1 and 1000 CE. Modeled results show that isotopic ranges for both sexes greatly overlap. The temporal plots show relatively constant δ^13^C values and some variability in δ^15^N values although there is an overall decrease after c. 500 CE. This likely reflects a combination of factors, including the end of the Roman proto-welfare system (*Annona* i.e. the yearly distribution of grain in Rome, which at times included pork^99^) and a reduction in the proportion of consumed pork in favor of ovicaprids as revealed by zooarchaeological studies^89,90,100^.

**Fig. 6:**
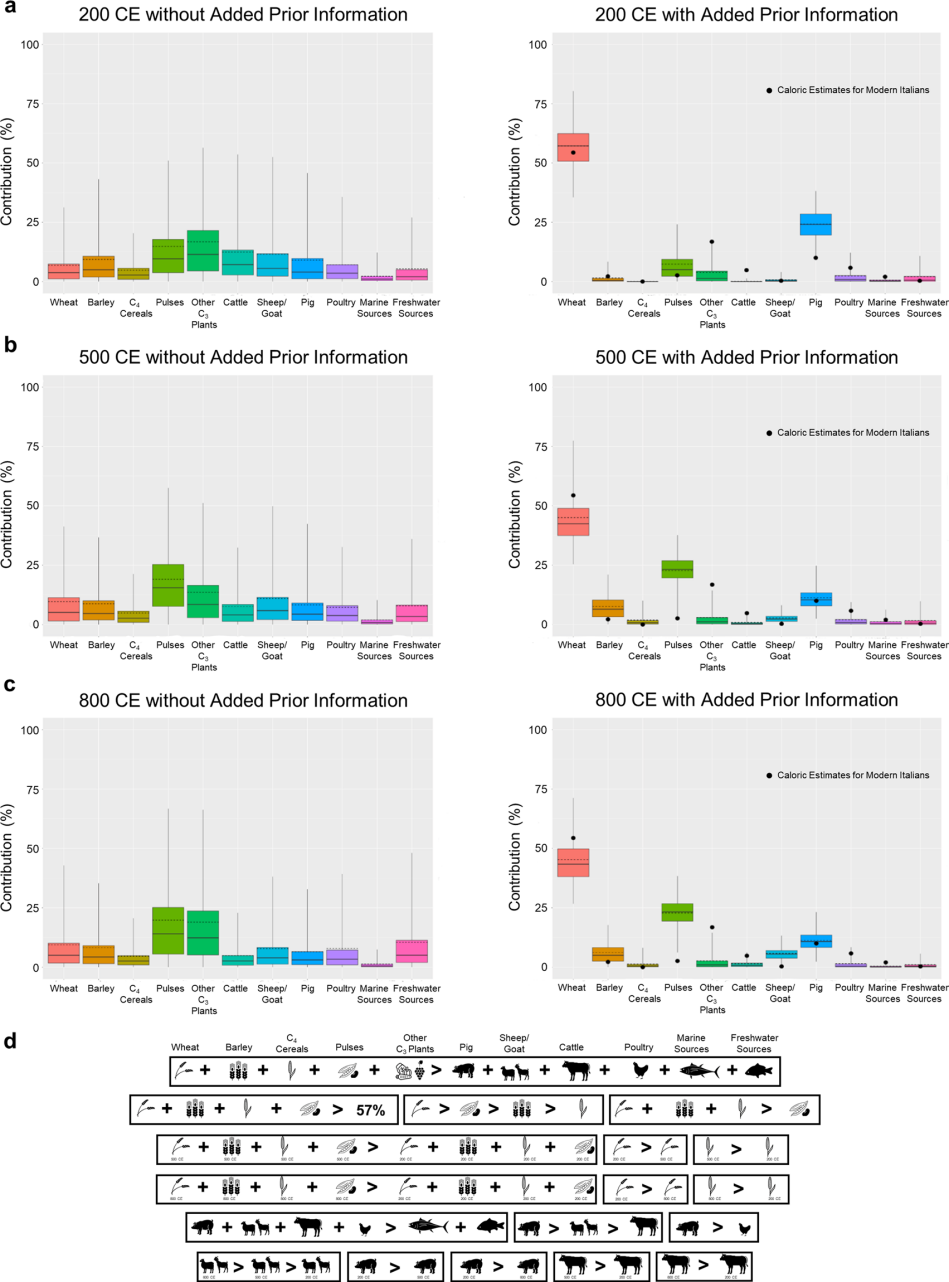
200 CE (**a**), 500 CE (**b**), and 800 CE (**c**) dietary estimated models of main food sources caloric contribution for the city of Rome without (left) and with (right) added prior dietary information (**d**). Black circles within plots correspond to modern dietary estimates. See also Supplementary Information Files S4–S6 for modeling details.

The study of medieval diets is also explored using a variety of nonisotopic evidence (e.g. written sources, archaeofaunal, and archaeobotanical studies). The integration of these types of evidence using Bayesian methods allows for improvements in the precision of dietary reconstructions^101,102^. Figure 6 shows the comparison of Bayesian dietary estimates for three separate time slices (200, 500, and 800 CE). It also includes a comparison of modeling relying only on isotopic data and vague priors (left) and of modeling combining isotopic data with non-isotopic dietary prior constraints obtained from ethnographic, archaeofaunal, archaeobotanical, and ancient textual studies (right) (modeling details and results in Supplementary Information File S4–S6). Clearly the use of isotopic data alone does not allow for precise dietary estimates given the uncertainties in model parameters and issues of equifinality (varying proportions of food intakes resulting in the same human isotopic value). Instead, the incorporation of prior dietary information^102^ resulted in a clear improvement in dietary precision that revealed diachronic trends and allowed for comparisons with modern day diets (Fig. 6, right, and Supplementary Information File S6).

**Fig. 7:**
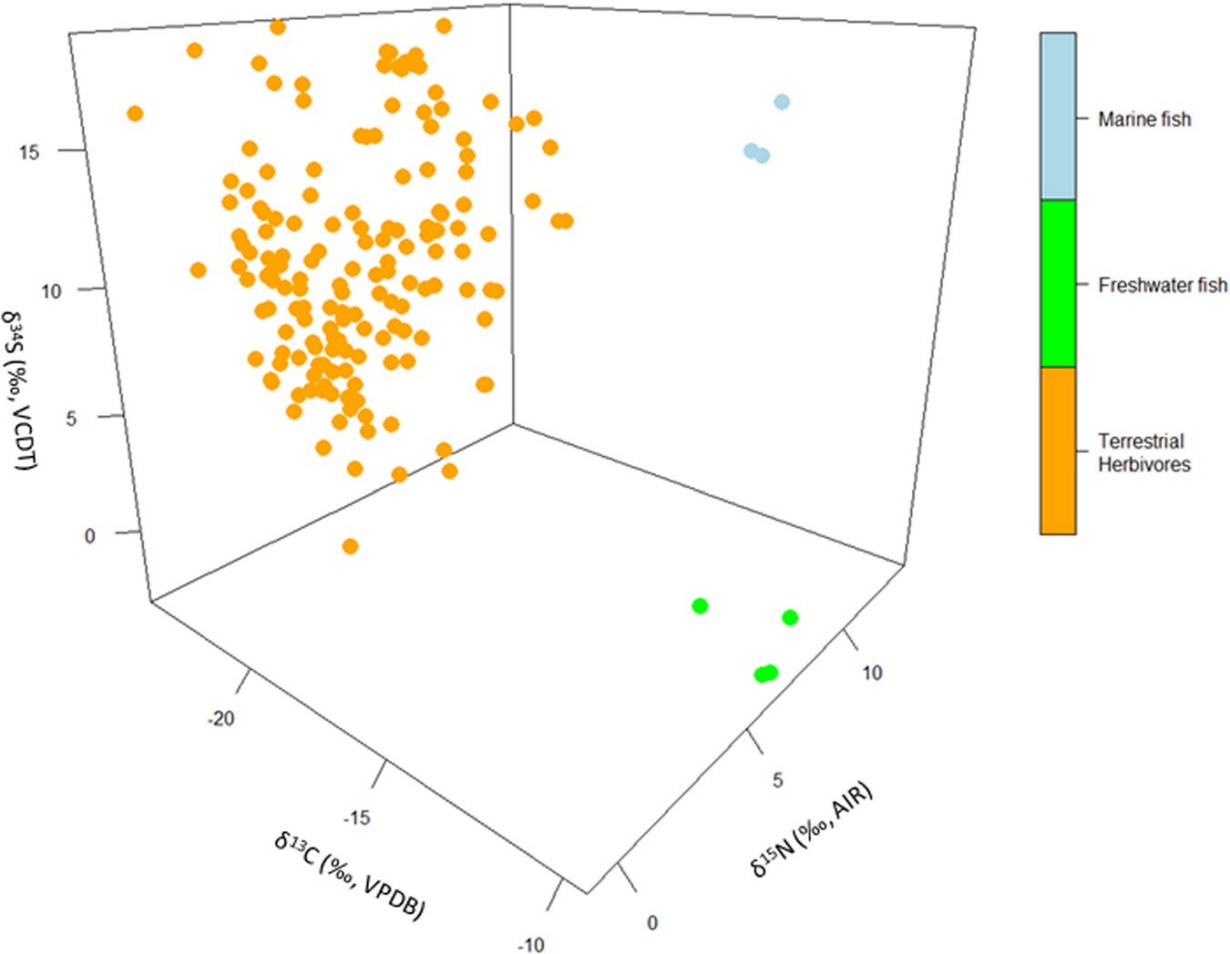
Distribution of δ^34^S, δ^15^N, and δ^13^C measurements on bone collagen included in CIMA from terrestrial herbivores, freshwater fish, and marine fish that passed elemental quality criteria (atomic ratios of C:N, C:S, and N:S).

The precision of modeled dietary estimates, and of other past phenomena, may also be improved by integrating data from multiple isotopic proxies. CIMA includes data from several isotopic proxies measured on human remains. Among these, are sulfur isotopic measurements (δ^34^S) that can exhibit a large spatial and environmental variability^103^. Figure 7 shows the distribution of δ^34^S, δ^15^N, and δ^13^C measurements on bone collagen included in CIMA from terrestrial herbivores, freshwater fish, and marine fish that passed elemental quality criteria (atomic ratios of C:N, C:S, and N:S)^71,73^. For the available data, the multi-proxy approach exhibits a clear separation among the taxa. However, the number of measurements available for marine fish (n = 3) and freshwater fish (n = 4) is small and lack representativity of the expected isotopic range. For instance, freshwater fish δ^13^C values are atypically high, values closer to terrestrial herbivores would be expected, and all originate from an Icelandic volcanic lake^104^. Unavailable from publications, were records for δ^34^S measurements in plants although these should be similar to those from collocated herbivores. These examples illustrate another important function of CIMA, to identify data gaps and set future research targets.

Isotopic data can also be employed to investigate human and animal spatial mobility patterns although here we only illustrate the former. In this respect, medieval Europe witnessed several population movements at various scales, from the mass migrations of the Germanic Migration Period (conventional 375–568 CE), to comparatively smaller scale movements following military conflicts, urbanization processes, and religious pilgrimages^1,6–8^. Isotopic studies of human mobility often explore spatial variability of water strontium (^87^Sr/^86^Sr) or oxygen (δ^18^O) isotopic ratios^17,105–108^. These can then be compared with measurements in human tissues with varying formation periods and turnover rates^53,55^.

**Fig. 8:**
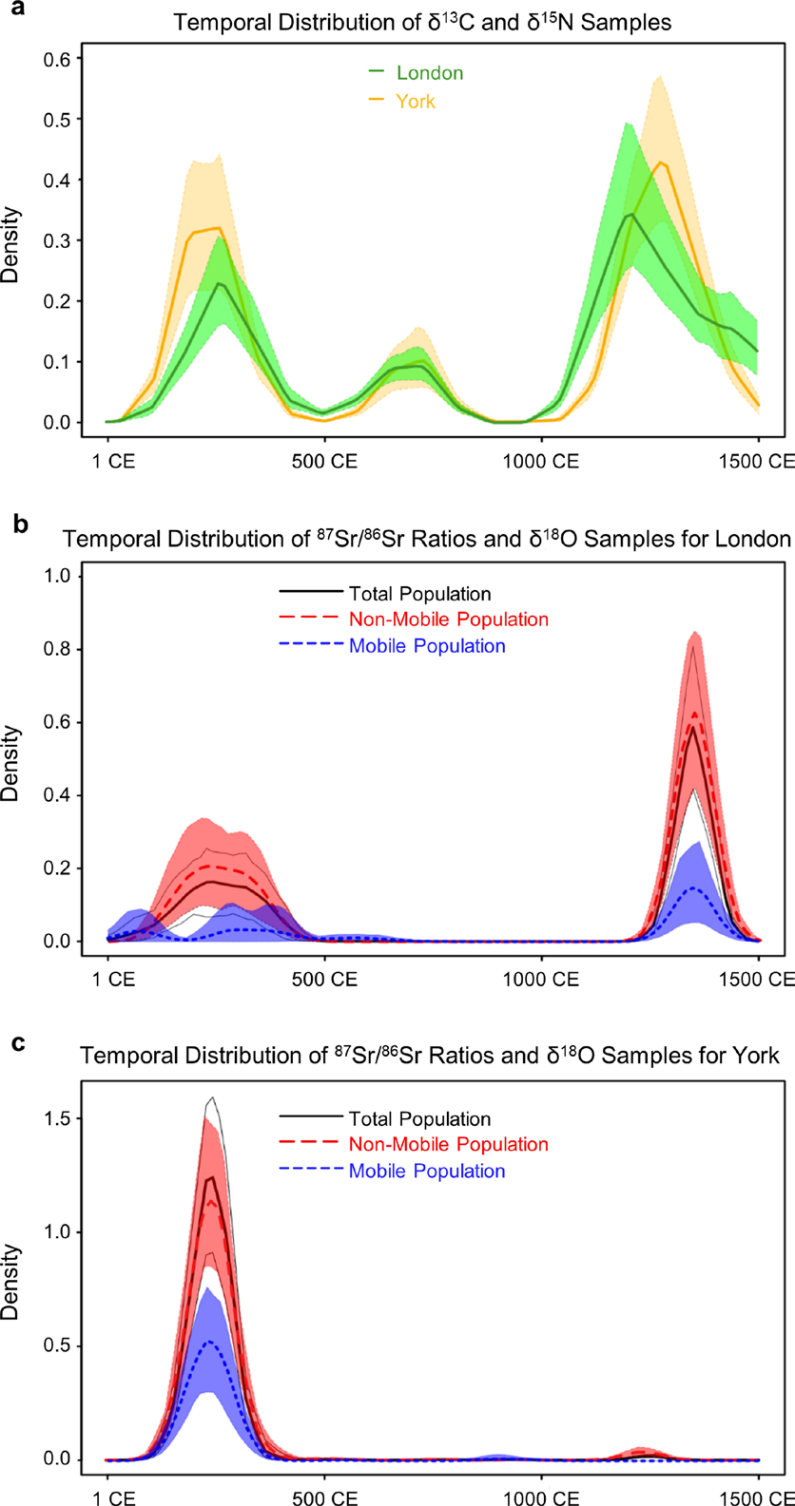
Kernel density plots for human osteological samples from London and York. Heights reflect relative temporal abundance of samples with δ^13^C and δ^15^N measurements (a -yellow and green plots), of ^87^Sr/^86^Sr or δ^18^O measurements (white plots), and of mobile (blue plots) and non-mobile (red plots) individuals.

Most common isotope-based mobility studies determine if investigated individuals have isotopic signatures matching burial locations. We illustrate this for Roman and medieval individuals buried at sites in York and London. Their ^87^Sr/^86^Sr or δ^18^O isotopic values measured on teeth are compared with a Bayesian reference baseline (modeling details in Supplementary Information File S4, S5). Individuals for which the values for one of these proxies did not match local values (overlap in 95% credible ranges) were classified as mobile or otherwise as non-mobile. Kernel density plots were then used to reveal the proportion of mobile vs. non-mobile individuals at each location (Fig. 8b,c). Also included in Fig. 8(a) are Kernel density plots for δ^13^C and δ^15^N bone/tooth collagen IRMS measurements for the same locations. These are more abundant than ^87^Sr/^86^Sr or δ^18^O and may reveal data gaps resulting not only from sampling bias but also from sample availability which depends on past population numbers, burial practices, and taphonomic effects. The modeling results show the presence of mobile individuals at both London and York across the periods for which data is available. However, variations in height ratios of mobile *versus* non-mobile individuals reveal that in London there was a comparatively higher proportion of mobile individuals during the early Roman Period and during the continental migration of the fifth century CE.

**Fig. 9:**
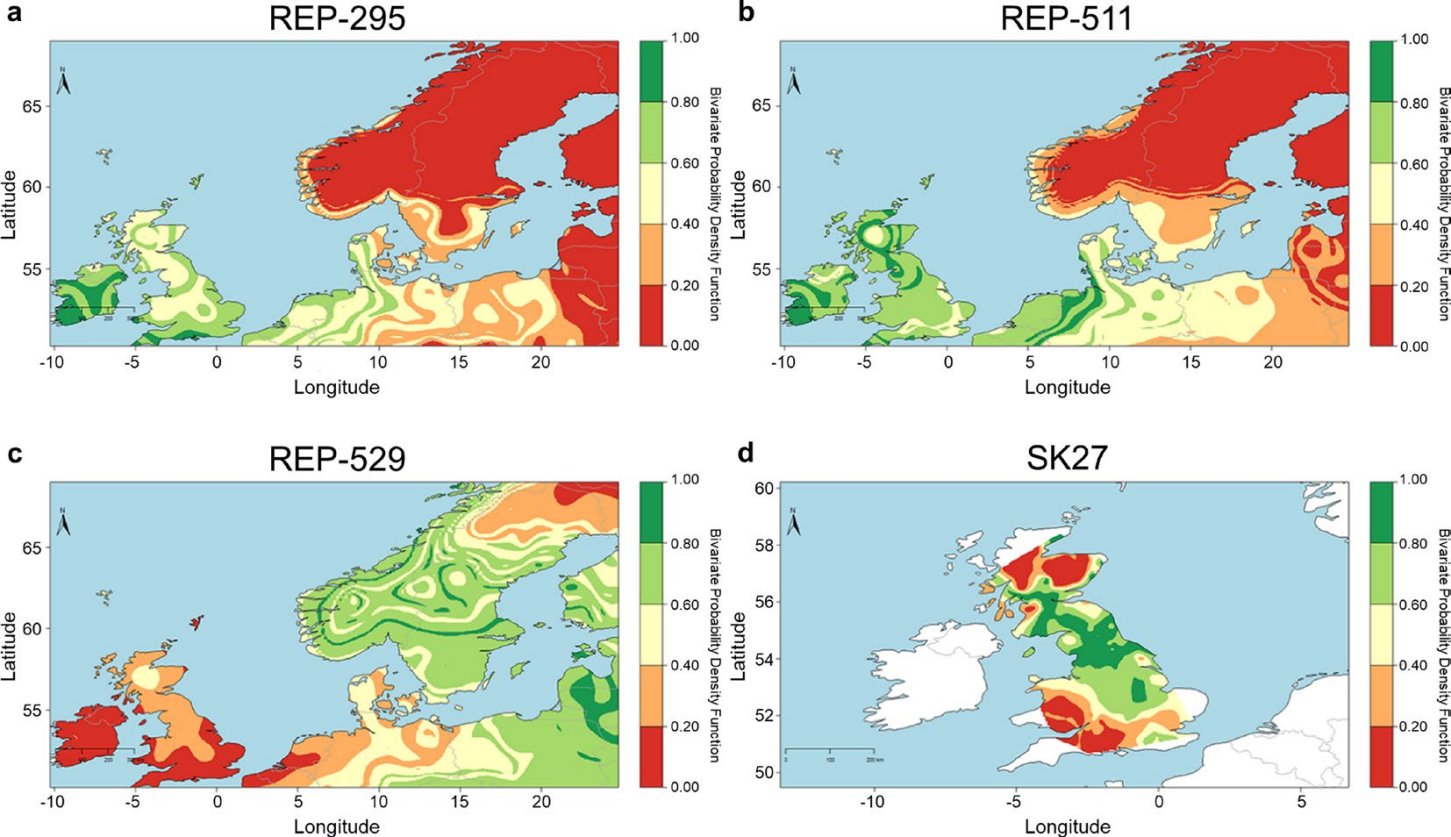
Probability density maps for place of origin for individuals REP-295, REP-511, REP-529 and SK27.

In some cases it may be possible to determine the place of origin of a mobile individual by comparing ^87^Sr/^86^Sr and/or δ^18^O values measured in a tooth formed at an early age with isotopic reference maps^61^. This assumes low mobility during the formation of the tooth and that the research areas may be somewhat constrained. Relying on δ^18^O tooth data we employ this approach to estimate the place of origin for three individuals (REP-295, REP-511, REP-529) buried in Repton, UK and associated to the Scandinavian ‘Great Heathen Army’, invading Britain in the late ninth century^109–112^. Modeling results (Fig. 9) show that individuals from a double grave (REP-295 and REP-511) likely originated from Ireland, which is often associated with campaigns led by some of the leaders of the army, although other regions in the British Isles and the opposing continental coast are also possible. On the other hand, the remaining Repton individual (REP-529) was likely from Sweden, Norway, or the Baltic region.

We also investigated the place of origin of a young individual (SK27), buried in a high medieval leprosarium in Winchester, UK, together with a scallop shell typical of a pilgrim who completed a pilgrimage to Santiago de Compostela, was determined using both ^87^Sr/^86^Sr and/or δ^18^O measurements. This individual was likely residing in northern England or in southern Scotland during tooth formation in accordance with previous reports (Fig. 9)^113^.

## Supplementary information

Supplementary Information File 1

Supplementary Information File 2

Supplementary Information File 3

Supplementary Information File 4

Supplementary Information File 5

Supplementary Information File 6

## Data Availability

The statistical analysis and modeling employed for examples given in the Usage Notes was done in R^80^ and included R packages developed within the Pandora & IsoMemo initiatives^59–61,82,83^. Source code for spatiotemporal models (AverageR, TimeR, OperatoR, KernelTimeR, and LocateR) is available for download at GitHub (https://github.com/Pandora-IsoMemo/iso-app) together with the source code for ReSources (https://github.com/Pandora-IsoMemo/resources). These can be run locally (https://github.com/Pandora-IsoMemo/drat) as Shiny apps^81^. For modeling reproducibility, a full description of model options is given in Supplementary Information S5. The MATILDA data community where CIMA is stored is part of the Pandora data platform that is based on the CKAN open source data management system (https://ckan.org/). This is hosted by the Max Planck Computing and Data Facility.
