# Supplementary material for: Presenting the Compendium Isotoporum Medii Aevi, a Multi-Isotope Database for Medieval Europe
Source: Sci Data. 2022 Jun 21;9:354. doi: 10.1038/s41597-022-01462-8 (PMC9213510; doi:10.1038/s41597-022-01462-8)
Supplement: Supplementary file 2 — Supplementary Information File 2 [file 41597_2022_1462_MOESM2_ESM.docx]

**Supplementary Information File S2: CIMA Bibliography**

Åberg, G., Fosse, G., Stray, H. (1998). Man, nutrition and mobility: A comparison of teeth and bone from the Medieval era and the present from Pb and Sr isotopes. *The Science of the Total Environment* **224**: 109-119.

Åborg, D.C. (2013). *Hierarchy through Diet. Stable isotope analysis of male graves of the estate church graveyard in Varnhem*. Unpublished BA dissertation: Stockholm University.

Aguraiuja-Lätti, Ü., Lõugas, L. (2019). Stable isotope evidence for medieval diet in urban and rural northern Estonia. *Journal of Archaeological Science: Reports* **26**: 101901. DOI: 10.1016/j.jasrep.2019.101901

Alaica, A.K., Schalburg-Clayton, J., Dalton, A., Kranioti, E., Graziani Echávarri, G., Pickard, C. (2019). Variability along the frontier: stable carbon and nitrogen isotope ratio analysis of human remains from the Late Roman–Early Byzantine cemetery site of Joan Planells, Ibiza, Spain. *Archaeological and Anthropological Sciences* **11**: 3783–3796.

Alexander, M.M., Gerrard, C.M., Gutiérrez, A., Millard, A.R. (2015). Diet, Society, and Economy in Late Medieval Spain: Stable Isotope Evidence from Muslims and Christians from Gandía, Valencia. *American Journal of Physical Anthropology* **156**: 263-273.

Alexander, M.M., Gutiérrez, A., Millard, A.R., Richards, M.P., Gerrard, C.M. (2019). Economic and socio-cultural consequences of changing political rule on human and faunal diets in medieval Valencia (c. fifth–fifteenth century AD) as evidenced by stable isotopes. *Archaeological and Anthropological Sciences* **11**: 3875–3893.

Al-Shorman, A., El-Khouri, L. (2011). Strontium isotope analysis of human tooth enamel from Barsinia: a late antiquity site in Northern Jordan. *Archaeological and Anthropological Sciences* **3**: 263-269.

Alt, K.W., Knipper, C., Peters, D., Müller, W., Maurer, A.F., Kollig, I., Nicklisch, N., Müller, C., Karimnia, S., Brandt, G., Roth, C., Rosner, M., Mende, B., Schöne, B.R., Vida, T., von Freeden, U. (2014). Lombards on the Move – An Integrative Study of the Migration Period Cemetery at Szólád, Hungary. *PLoS ONE* **9**: e110793. DOI: 10.1371/journal.pone.0110793.

Amorim, C.E.G., Vai, S., Posth, C., Modi, A., Koncz, I., Hakenbeck, S., La Rocca, M.C., Mende, B., Bobo, D., Pohl, W., Pejrani Baricco, L., Bedini, E., Francalacci, P., Giostra, C., Vida, T., Winger, D., von Freeden, U., Ghirotto, S., Lari, M., Barbujani, G., Krause, J., Caramelli, D., Geary, P.J., Veeramah, K.R. (2018). Understanding 6th-century barbarian social organization and migration through paleogenomics. *Nature Communications* **9**. DOI: 10.1038/s41467-018-06024-4.

Arneborg, J., Heinemeier, J., Lynnerup, N., Nielsen, H.L., Rud, N., Sveinbjornsdottir, Á.E. (1999). Change of diet of the Greenland Vikings determined from stable carbon isotope analysis and 14C dating of their Bones. *Radiocarbon* **41**: 157-168.

Arneborg, J., Lynnerup, N., Heinemeier, J. (2012). Human Diet and Subsistence Patterns in Norse Greenland AD C.980—AD c. 1450: Archaeological interpretations. *Journal of the North Atlantic* **3**: 119-133.

Ascough, P.L., Cook, G.T., Church, M.J., Dugmore, A.J., McGovern, T.H., Dunbar, E., Einarsson, Á., Friðriksson, A., Gestsdóttir, H. (2007). Reservoirs and radiocarbon: 14C dating problems in Mývatnssveit, Northern Iceland. *Radiocarbon* **49**: 947-961.

Ascough, P.L., Cook, G.T., Church, M.J., Dunbar, E., Einarsson, A., McGovern, T.H., Dugmore, A.J., Perdikaris, S., Hastie, H., Friðriksson, A., Gestsdóttir, H. (2010). Temporal and spatial variations in freshwater 14C reservoir effects: Lake Myvatn, Northern Iceland. *Radiocarbon* **52**: 1098-1112.

Ascough, P.L., Cook, G.T., Church, M.J., Dunbar, Gestsdóttir, H., McGovern, T.H., Dugmore, A.J., Friðriksson, A., Edwards, K.J. (2012). Radiocarbon reservoir effects in human bone collagen from northern Iceland. *Journal of Archaeological Science* **39**: 2261-2271.

Bäckström, Y., Mispelaere, J., Ingvarsson, A., Fjellström, M., Britton, K. (2018). Integrating isotopes and documentary evidence: dietary patterns in a late medieval and early modern mining community, Sweden. *Archaeological and Anthropological Sciences* **10**: 2075-2094.

Baldoni, M., Nardi, Müldner, G., Lelli, R., Gnes, M., Ferraresi, F., Meloni, V., Cerino, P., Greco, S., Manenti, G., Angle, M., Rickards, O., Martínez-Labarga, C. (2016). Archaeo-biological reconstruction of the Italian medieval population of Colonna (8th–10th centuries CE). *Journal of Archaeological Science: Reports* **10**: 483-494.

Baldoni, M., Scorrano, G., Alexander, M., Stasolla, F.R., Marsella, L.T., Rickards, O., Martínez-Labarga, C. (2019). The medieval population of Leopoli-Cencelle (Viterbo, Latium): Dietary reconstruction through stable isotope analysis from bone proteins. *Journal of Archaeological Science: Reports* **24**: 92-101.

Baldoni, M., Scorrano, G., Gismondi, A., D’Agostino, A., Alexander, M., Gaspari, L., Vallelonga, F., Canini, A., Rickards, O., Martínez-Labarga, C. (2018). Who were the miners of Allumiere? A multidisciplinary approach to reconstruct the osteobiography of an Italian worker community. *PLoS ONE* **13**: e0205362. DOI: 10.1371/journal.pone.0205362.

Barrett, J.H., Boessenkool, S., Kneale, C.J., O’Connell, T.C. (2020). Ecological globalisation, serial depletion and the medieval trade of walrus rostra. *Quaternary Science Reviews* **229**: 106122. DOI: 10.1016/j.quascirev.2019.106122.

Barrett, J.H., Johnstone, C., Harland, J., Van Neer, W., Ervynck, A., Makowiecki, D., Heinrich, D., Hufthammer, A.K., Enghoff, I.B., Amudsen, C., Christiansen, J.S., Jones, A.K.G., Locker, A., Hamilton-Dyer, S., Jonsson, L., Lõugas, L., Roberts, C., Richards, M.P. (2008). Detecting the medieval cod trade: a new method and first results. *Journal of Archaeological Science* **35**: 850-861.

Barrett, J.H., Orton, D., Johnstone, C., Harland, J., Van Neer, W., Ervynck, A., Roberts, C., Locker, R., Amundsen, C., Bødker Enghoff, I., Hamilton-Dyer, S., Heinrich, D., Hufthammer, A.K., Jones, A.K.G., Jonsson, L., Makowiecki, D., Pope, P., O’Connell, T.C., de Roo, T., Richards, M.P. (2011). Interpreting the expansion of sea fishing in medieval Europe using stable isotope analysis of archaeological cod bones. *Journal of Archaeological Science* **38**: 1516-1524.

Barrett, J.H., Richards, M.P. (2004). Identity, gender, religion and economy: New isotope and radiocarbon evidence for marine resource intensification in early historic Orkney, Scotland, UK. *European Journal of Archaeology* **7**: 249-271.

Bayliss, A., Sheperd Popescu, E., Beavan-Athfield, N., Bronk Ramsey, C., Cook, G.T., Locker, A. (2004). The potential significance of dietary offsets for the interpretation of radiocarbon dates: an archaeologically significant example from medieval Norwich. *Journal of Archaeological Science* **31**: 563-575.

Beaumont, J., Bekvalac, J., Harris, S., Batt, C.M. (2021). Identifying cohorts using isotope mass spectrometry: the potential of temporal resolution and dietary profiles. Archaeometry. DOI: 10.1111/arcm.12667

Beaumont, J., Craig-Atkins, E., Buckberry, J., Haydock, H., Horne, P., Howcroft, R., Mackenzie, K., Montgomery J. (2018). Comparing apples and oranges: Why infant bone collagen may not reflect dietary intake in the same way as dentine collagen. *American Journal of Physical Anthropology* **167**: 524-540.

Beaumont, J. Gledhill, A., Montgomery, J. (2014). Isotope analysis of incremental human dentine: towards higher temporal resolution. *Bulletin of the International association for paleodontology* **8**: 212-223.

Beavan-Athfield, N. and Mays, S. (2009). Amino-acid and stable-isotope analysis: investigation of bone protein survival and dietary implications. In Scull, C. (ed.). *Early medieval (late 5th-early 8th centuries AD) cemeteries at Boss Hall and Buttermarket, Ipswich, Suffolk*. Leeds. 222-226.

Bliujienė, A., Skipitytė, R., Garbaras, A. Miliauskienė, A., Šapolaitė, J., Ežerinskis, Z., Čeponkus, J., Masiulienė, I., Simčenka, E., Minkevičius, K., Piličiauskienė, G. (2020). The first data on the human diet in Late Roman and Early Migration period western Lithuania: Evidence from stable isotope, archaebotanical and zooarchaeological analyses. *Journal of Archaeological Science: Reports* **33**: 102545. DOI: 10.1016/j.jasrep.2020.102545

Bocherens, H., Fizet, M., Mariotti, A., Olive, C., Bellon, G., Billiou, D. (1991). Application de la biogeochimie isotopique (13C, 15N) a la déetermination du régime alimentaire des populations humains et animales durante les périodes antique et médiévale. *Archives des Sciences – Université de Genève* **44**: 329-340.

Boudin, M., Boeckx, P., Vandenabeele, P., Van Strydock, M. (2014). An Archaeological mystery revealed by radiocarbon dating of cross-flow nanofiltrated amino acids derived from bone collagen, silk and hair: Case study of the bishops Baldwin I and Radbot II from Noyon-Tournai. *Radiocarbon* **56**: 603-617.

Bourbou, C., Fuller, B.T., Garvie-Lok, S.J., Richards, M.P. (2011). Reconstructing the Diets of Greek Byzantine Populations (6th–15th Centuries AD) Using Carbon and Nitrogen Stable Isotope Ratios. *American Journal of Physical Anthropology* **146**: 569-581.

Bourbou, C., Fuller, B.T., Garvie-Lok, S.J., Richards, M.P. (2013). Nursing mothers and feeding bottles: reconstructing breastfeeding and weaning patterns in Greek Byzantine populations (6the15th centuries AD) using carbon and nitrogen stable isotope ratios. *Journal of Archaeological Science* **40**: 3903-3913.

Bourbou, C., Richards, M.P. (2007). The Middle Byzantine Menu: Palaeodietary Information from Isotopic Analysis of Humans and Fauna from Kastella, Crete. *International Journal of Osteoarchaeology* **17**: 63-72.

Bownes, J., Clarke, L., Buckberry, J. (2018). The importance of animal baselines: Using isotope analysis to compare diet in a British medieval hospital and lay population. *Journal of Archaeological Science: Reports* **17**: 103-110.

Brettell, R., Evans, J., Marzinzik, S., Lamb, A., Montogomery, J. (2012). ‘Impious Easterners’: Can Oxygen and Strontium Isotopes Serve as Indicators of Provenance in Early Medieval European Cemetery Populations?. *European Journal of Archaeology* **15**: 117-145.

Britton, K., Fuller, B.T., Tütken, T., Mays, S., Richards, M.P. (2015). Oxygen Isotope Analysis of Human Bone Phosphate Evidences Weaning Age in Archaeological Populations. *American Journal of Physical Anthropology* **157**: 226-241.

Brozou, A., Lynnerup, N., Mannino, M.A., Millard, A.R., Gröcke, D.R. (2019). Investigating dietary patterns and organisational structure by using stable isotope analysis: a pilot study of the Danish medieval leprosy hospital at Næstved. *Anthropologischer Anzeiger* **76**: 167-178.

Buchan, M., Müldner, G., Ervynck, A., Britton, K. (2015). Season of birth and sheep husbandry in late Roman and Medieval coastal Flanders: A pilot study using tooth enamel δ18O analysis. *Environmental Archaeology* **21**: 260-270.

Budd, P., Chenery, C., Montgomery, J., Evans, J., Powlesland, D. (2003). Anglo-Saxon residential mobility at West Heslerton, North Yorkshire, UK from combined O- and SR-isotope analysis. In Greenville, H., Scott, D.T. (eds.), *Plasma Source Mass Spectometry. Applications and Emerging Technologies*. ‘Proceedings of the 8th International Conference on Plasma Source Mass Spectometry, University of Durham, 8-13 September 2002’. Cambridge. 195-208.

Budd, P., Millard, A., Chenery, C., Lucy, S., Roberts, C. (2004). Investigating population movement by stable isotope analysis: a report from Britain. *Antiquity* **78**: 127-141.

Budd, P., Montgomery, J., Barreiro, B., Thomas, R.G. (2000). Differential diagenesis of strontium in archaeological human dental tissues. *Applied Geochemistry* **15**: 687-694.

Buonincontri, M.P., Pecci, A., Di Pasquale, G., Ricci, P., Lubritto, C. (2017). Multiproxy Approach to the study of Medieval food habits in Tuscany (central Italy). *Archaeological and Anthropological Science* **9**: 653-671.

Burrell, C.L., Emery, M.M., Gonzales, S. (2019). Paget's disease of bone in two medieval skeletons from Poulton Chapel, Cheshire, UK. *International Journal of Osteoarchaeology* **29**: 922-933.

Burt, N.M. (2013). Stable Isotope Ratio Analysis of Breastfeeding and Weaning Practices of Children from Medieval Fishergate House York, UK. *American Journal of Physical Anthropology* **152**: 407-416.

Cahill Wilson, J., Standish, C.D. (2016). Mobility and migration in late Iron Age and early Medieval Ireland. *Journal of Archaeological Science: Reports* **6**: 230-241.

Canavan, S.M., Burrell, C.L., Emery, M.M., Gonzales, S. (2019). A case of projectile trauma from the medieval Poulton Chapel, Cheshire, the United Kingdom. *International Journal of Osteoarchaeology* **29**: 1079-1090.

Cau, M.Á., Rullan, M.R., Salas, M., Van Strydonck, M. (2014). Radiocarbon dating of the necropolis of Early Christian Site of Son Peretó (Mallorca, Balearic Islands). *Radiocarbon* **56**: 399-410.

Chenery, C.A., Evans, J.A., Score, D., Boyle, A., Chenery, S.R. (2014). A Boat Load of Vikings?. *Journal of the North Atlantic* **7**: 43-53.

Ciaffi, R., Lelli, R., Müldner, G. Stantcheva, K., Fischetti, A.L., Ghini, G., Craig, O.E., Milano, F. Rickards, O., Arcudi, G., Martínez-Labarga, C. (2015). Palaeobiology of the Medieval population of Albano (Rome, Italy): A combined morphological and biomolecular approach. *International Journal of Osteoarchaeology* **25**: 477-488.

Cole, Z.A. (2019). *Fish, Oats & Isotopes. An Overview of Stable Isotopic Research on Human Diet in Medieval Scandinavia c. 1050-1550 CE*. Unpublished MA dissertation: University of Iceland.

Colleter, R., Clâvel, B., Pietrzak, A., Duchesne, S., Schmitt, L., Richards, M.P., Telmon, N., Crubézy, É., Jaouen, K. (2019). Social status in late medieval and early modern Brittany: insights from stable isotope analysis. *Archaeological and Anthropological Sciences* **11**: 823-837.

Craig-Atkins. E., Jervis, B., Cramp, L., Hammann, S., Nederbragt, A.J., Nicholson, E., Taylor, A.R., Whelton, H., Madwick, R. (2020). The dietary impact of the Norman Conquest: A multiproxy archaeological investigation of Oxford, UK. *PLoS ONE* **15**: e0235005. DOI: 10.1371/journal.pone.0235005

Craig-Atkins, E., Towers, J., Beaumont, J. (2018). The role of infant life histories in the construction of identities in death: An incremental isotope study of dietary and physiological status among children afforded differential burial. *American Journal of Physical Anthropology* **167**: 644-655.

Croix, S., Frei, K.M., Sindbæk, S.M., Søvsø, M. (2020). Individual geographic mobility in a Viking-Age emporium—Burial practices and strontium isotope analyses of Ribe’s earliest inhabitants. *PLoS ONE* **15**: e0237850. DOI: 10.1371/journal.pone.0237850

Crowder, K.D., Montgomery, J., Filipek, K.L., Evans, J.A. (2020). Romans, barbarians and foederati: New biomolecular data and a possible region of origin for “Headless Romans” and other burials from Britain. *Journal of Archaeological Science: Reports* **30**: 102180. DOI: 10.1016/j.jasrep.2019.102180

Crowder, K.D., Montgomery, J., Gröcke, D.R., Filipek, K.L. (2019). Childhood “stress” and stable isotope life histories in Transylvania. *International Journal of Osteoarchaeology* **29**: 644-653.

Curtis-Summer, S., Montgomery, J., Carver, M. (2014). Stable Isotope Evidence for Dietary Contrast Between Pictish and Medieval Populations at Portmahomack, Scotland. *Medieval Archaeology* **58**: 21-43.

Curtis-Summer, S., Pearson, J.A., Lamb., A.L. (2020). From Picts to Parish: Stable isotope evidence of dietary change at medieval Portmahomack, Scotland. *Journal of Archaeological Science: Reports* **102303**. DOI: 10.1016/j.jasrep.2020.102303

Curto, A., Mahoney, P., Maurer, A.F., Barrocas-Dias, C., Fernandes, T., Fahy, G.E. (2019). Diet and disease in Tomar, Portugal: Comparing stable carbon and nitrogen isotope ratios between skeletons with and without signs of infectious disease. *Journal of Archaeological Science* **105**: 59-69.

Curto, A., Maurer, A.F., Barrocas-Dias, C., Mahoney, P., Fernandes, T., Fahy, G.E. (2019). Did military orders influence the general population diet? Stable isotope analysis from Medieval Tomar, Portugal. *Archaeological and Anthropological Sciences* **11**: 3797–3809.

Czermak, A., Schermelleh, L., Lee-Thorp, J. (2018). Imaging-assisted time-resolved dentine sampling to track weaning histories. *International Journal of Osteoarchaeology* **28**: 535-541.

Czermak, A., Schermelleh, L., Lee-Thorp, J. (2019). Fluorescence screening of collagen preservation in tooth dentine. *Palaeogeography, Palaeoclimatology, Palaeoecology* **532**: 109249. DOI: 10.1016/j.palaeo.2019.109249

Daux, V., Lécuyer, C., Adam, F., Martineau, F., Vimeux, F. (2005). Oxygen isotope composition of human teeth and the record of climate changes in France (Lorraine) during the last 1770 years. *Climatic Change* **70**: 445-464.

Dobrovolskaya, M.V., Tiunov, A.V., Krylovich, O.A., Kuzmicheva, E.A., Reshetova, I.K., Savinetsky, A.B., Svirkina, N.G., Smirnov, A.L., (2020). Isotope markers of ecosystems and nutrition of the medieval rural population in the forest zone of European Russia. *Rossiiskaia arkheologiia* **3**: 79-95.

Dotsika, E., Michael, D.E., Iliadis, E., Karalis, P., Diamantopoulos, G. (2018). Isotopic reconstruction of diet in Medieval Thebes (Greece). *Journal of Archaeological Science: Reports* **22**: 482-491.

Dreshaj, M. (2017). *Study of paleodiet from the context of the rotunda church in Bribirska Glavica, Croatia*. Unpublished MA dissertation: University of Evora.

Dreslerová. D., Hajnalová, M., Trubač, J., Chuman, T., Kočár, P., Kunzová, E., Šefrna, L. (2020). Maintaining soil productivity as the key factor in European prehistoric and medieval farming. *Journal of Archaeological Science: Reports* **35**: 102633. DOI: 10.1016/j.jasrep.2020.102633

Duignan, S.E. (2015). *A Tale of Two Isotopes: Exploring Human Movement through Strontium Isotope Analysis in Two Medieval Danish Cemetery Populations.* Unpublished MA dissertation: University of Manitoba.

Dury, G., Eriksson, G., Fjellström, M., Wallerström, T., Lidén, K. (2018). Consideration of freshwater and multiple marine reservoir effects: Dating of individuals with mixed diets from northern Sweden. *Radiocarbon* **60**: 1561-1585.

Dury, G., Lythe, A., Marquez-Grant, N., Garcia-Rubio, A., Graziani, G., Mari, J., Ziriax, M., Schulting, R. (2019). The Islamic cemetery at 33 Bartomeu Vicent Ramon, Ibiza: investigating diet and mobility through light stable isotopes in bone collagen and tooth enamel. *Archaeological and Anthropological Sciences* **11**: 3913–3930.

Edwards, K.J., Cook, G.T., Nyegaard, G., Schofield, J.E. (2013). Towards a first chronology for the middle settlement of Norse Greenland: 14C and related studies of animal bone and environmental material. *Radiocarbon* **55**: 13-29.

Ervynck, A., Boudin, M., van den Brande, T., Van Strydonck, M. (2014). Dating human remains from the historical period in Belgium: Diet changes and the impact of marine and freshwater reservoir effects. *Radiocarbon* **56**: 779-788.

Ervynck, A., Deckers, P., Lentacker, A., Tys, D., Van Neer, W. (2012). ‘Leffinge - Oude Werf’: the first archaeozoological collection from a *terp* settlement in coastal Flanders. In Raemaekers, D.C.M., Esser, E., Lauwerier, R.C.G.M., Zeiler, J.T. (eds.), *A Bouquet of Archaeological Studies. Essays in Honour of Wietske Prummel*. Groningen. 151-160.

Ervynck, A., Lentacker, A., Müldner, G., Richards, M.P., Dobney, K. (2007). An investigation into the transition from forest dwelling pigs to farm animals in Medieval Flanders, Belgium. In Albarella, U., Dobney, K., Ervynck, A., Rowley-Conwy, P. (eds.), *Pigs and Humans. 10000 Years of Interaction*. Oxford. 171-193.

Evans, J.A., Tatham, S. (2004). Defining 'local signature' in terms of Sr isotope composition using a tenth- to twelfth-century Anglo-Saxon population living on a Jurassic clay-carbonate terrain, Rutland, UK. In Pye, K., Croft, D. J. (eds), *Forensic Geoscience: Principles, Techniques and Applications*. London. 237-248.

Evans, J.A., Tatham, S., Chenery, S.R., Chenery, C.A. (2007). Anglo-Saxon animal husbandry techniques revealed though isotope and chemical variations in cattle teeth. *Applied Geochemistry* **22**: 1994-2005.

Fahy, G.E., Deter, C., Pitfield, R., Miszkiewicz, J.J., Mahoney, P. (2017). Bone deep: Variation in stable isotope ratios and histomorphometric measurements of bone remodelling within adult humans. *Journal of Archaeological Science* **87**: 10-16.

Farbes, E., Rose, A., Lee-Thorp, J., Loe, L., Hamerow, H. (2017). Temporal Trends in Medieval Diet at Stoke Quay, Ipswich, England. *American Journal of Physical Anthropology* **162**. Poster.

Fernandes, D., Sirak, K., Cheronet, O., Howcroft, R., Čavka, M., Los, D., Burmaz, J., Pinhasi, R., Novak, M. (2019). Cranial deformation and genetic diversity in three adolescent male individuals from the Great Migration Period from Osijek, eastern Croatia. *PLoS ONE* **14**: e0216366. DOI: 10.1371/journal.pone.0216366.

Fernanez-Martinez, P., Maurer, A.F., Jimenez-Morillo, N.T., Botella, M., Lopez, B., Barrocas Dias, C. (2020). Bone stable isotope data of the Late Roman population (4^th^–7^th^ centuries CE) from Mondragones (Granada): A dietary reconstruction in a Roman villa context of south-eastern Spain. *Journal of Archaeological Science: Reports* **33**: 102566. DOI: 10.1016/j.jasrep.2020.102566.

Fetner, R.A., Iwaszczuk, U. (2020). Isotopic evidence of possible long-distance freshwater fish trade in the 13th to 14th century Chełm, modern Poland. *International Journal of Osteoarchaeology*. DOI: 10.1002/oa.2931

Filipe, V., Toso, A., Inocêncio, J. (2017). Perspectivas arqueobiológicas sobre a necrópole islâmica de Alfama. In Caessa, A., Nozes, C., Cameira, I., Banha da Silva, R. (ed.), *I Encontro de Arqueologia de Lisboa: Uma Cidade em Escavação*, ‘Teatro Aberto, 26, 27 e 28 de Nov. de 2015’. Lisbon. 339-347.

Fisher, A., Thomas, R. (2012). Isotopic and zooarchaeological investigation of later medieval and post-medieval cattle husbandry at Dudley Castle, West Midlands. *Environmental Archaeology* **17**: 151-167.

Fjellström, M., Eriksson, G., Lidén, K., Svestad, A. (2019). Food and Cultural Traits in Coastal Northern Finnmark in the 14th–19th Centuries. *Norwegian Archaeological Review* **52**: 20-40.

Francisci, G., Micarelli, I., Iacumin, P., Castorina, F., Di Vincenzo, F., Di Matteo, M., Giostra, C., Manzi, G., Tafuri, M.A. (2020). Strontium and oxygen isotopes as indicators of Longobards mobility in Italy: an investigation at Povegliano Veronese. *Nature. Scientific Reports* **10**: 11678. DOI:  10.1038/s41598-020-67480-x

Frémondeau, D., De Cupere, B., Evin, A., Van Neer, W. (2017). Diversity in pig husbandry fromthe Classical-Hellenistic to the Byzantine periods: An integrated dental analysis of Düzen Tepe and Sagalassos assemblages (Turkey). *Journal of Archaeological Science: Reports* **11**: 38-52.

Frolik, J., Sneberger, J., Svetlik, I., Kaupová, S.D., Brabcova, K.P., Ovsonkova, Z.A. (2020). The oldest rulers of early medieval Bohemia and radiocarbon data. *Radiocarbon*. DOI: 10.1017/RDC.2020.62

Fuller, B.T., De Cupere, B., Marinova, E., Van Neer, W., Waelkens, M., Richards, M.P. (2012). Isotopic Reconstruction of Human Diet and Animal Husbandry Practices During the Classical-Hellenistic, Imperial, and Byzantine Periods at Sagalassos, Turkey. *American Journal of Physical Anthropology* **149**: 157-171.

Fuller, B.T., Márquez-Grant, N., Richards, M.P. (2010). Investigation of Diachronic Dietary Patterns on the Islands of Ibiza and Formentera, Spain: Evidence from Carbon and Nitrogen Stable Isotope Ratio Analysis. *American Journal of Physical Anthropology* **143**: 512-522.

Fuller, B.T., Müldner, G., Van Neer, W., Ervynck, A., Richards, M.P. (2012). Carbon and nitrogen stable isotope ratio analysis of freshwater, brackish and marine fish from Belgian archaeological sites (1st and 2nd millennium AD). *Journal of Analytical Atomic Spectrometry* **27**: 807-820.

García-Collado, M.I. (2016). Food consumption patterns and social inequality in an early medieval rural community in the centre of the Iberian Peninsula. In Quirós Castillo, J.A. (ed.), *Social complexity in early medieval rural communities. The north-western Iberia archaeological record*. Oxford. 59-78.

García-Collado, M.I., Ricci, P., Catalán Ramos, R., Altieri, S., Lubritto, C., Quirós Castillo, J.A. (2019). Palaeodietary reconstruction as an alternative approach to poorly preserved early medieval human bone assemblages: the case of Boadilla (Toledo, Spain). *Archaeological and Anthropological Sciences* **11**: 3765–3782.

Garvie-Lok, S.J. (2001). *Loaves and fishes: a stable isotope reconstruction of diet in Medieval Greece*. Unpublished PhD dissertation: University of Calgary.

Garvie-lok, S.J. (2009). Population Mobility at Frankish Corinth: Evidence from Stable Oxygen Isotope Ratios of Tooth Enamel. *Hesperia Supplements* **43**: 245-256.

Gismondi, A., Baldoni, M., Gnes, M., Scorrano, G., D’Agostino, A., Di Marco, G., Calabria, G., Petrucci, M., Müldner, G., Von Tersch, M., Nardi, A., Enei, F., Canini, A., Rickards, O., Alexander, M., Martínez-Labarga, C. (2020). A multidisciplinary approach for investigating dietary and medicinal habits of the Medieval population of Santa Severa (7th-15th centuries, Rome, Italy). *PLoS One* **15**: e0227433. DOI: 10.1371/journal.pone.0227433.

Gough, H. (2013). *Isotopes and Teeth: Human Movement in two Medieval Danish Cemetery Populations*. Unpublished MA dissertation: University of Manitoba.

Gregoricka, L.A., Judd, M.A. (2015). Isotopic Evidence for Diet Among Historic Bedouin of Khirbat al-Mudayna, Jordan. *International Journal of Osteoarchaeology* **26**: 705-715.

Gregoricka, L.A., Sheridan, S.G. (2013). Ascetic or affluent? Byzantine diet at the monastic community of St. Stephen’s, Jerusalem from stable carbon and nitrogen isotopes. *Journal of Anthropological Archaeology* **32**: 63-73.

Gregoricka, L.A., Sheridan, S.G., Schirtinger, M. (2017). Reconstructing life histories using multi-tissue isotope analysis of commingled remains from St. Stephen’s Monastery in Jerusalem: Limitations and potential. *Archaeometry* **59**: 148-163.

Grimes, V., Fuller, B.T., Guiry, E.J. (2014). Reconstructing Diets and Origins of Vikings at Hrísbrú, Mosfell Valley, Iceland: The Carbon, Nitrogen, and Strontium Isotope Evidence. In Zori, D., Byock, J. (eds.), *Viking Archaeology in Iceland: Mosfell Archaeological Project*. Turnhout. 105-116.

Gron, K.J, Larsson, M., Gröcke, D.R., Andersen, N.H, Andreasen, M.H., Bech, J.H., Henriksen, P.S., Hilton, R.G., Jessen, M.D., Møller, N.A., Nielsen, F.O., Nielsen, P.O., Pihl, A., Sørensen, L., Westphal, J., Rowley-Conwy, P., Church, M.J. (2021). Archaeological cereals as an isotope record of long-term soil health and anthropogenic amendment in southern Scandinavia. *Quaternary Science Reviews* **253**: 106762. DOI: 10.1016/j.quascirev.2020.106762

Groves, S.E., Roberts, C.A., Lucy, S., Pearson, G., Gröcke, D.R., Nowell, G., Macpherson, C.G., Young, G. (2013). Mobility Histories of 7th–9th Century AD People Buried at Early Medieval Bamburgh, Northumberland, England. *American Journal of Physical Anthropology* **151**: 462-476.

Grumbkow, P.v., Frommer, S., Kootker, L.M., Davies, G.R., Mazanec, J., Hummel, S. (2013). Kinship and mobility in 11th-century A.D. Gammertingen, Germany: an interdisciplinary approach. *Journal of Archaeological Science* **40**: 3768-3776.

Grupe, G., von Carnap-Bornheim, C., Becker, C. (2013). Rise and Fall of a Medieval Trade Centre: Economic Change from Viking Haithabu to Medieval Schleswig Revealed by Stable Isotope Analysis. *European Journal of Archaeology* **16**: 137-166.

Grupe, G., Heinrich, D., Peters, J. (2009). A brackish water aquatic foodweb: trophic levels and salinity gradients in the Schlei fjord, Northern Germany, in Viking and medieval times. *Journal of Archaeological Science* **36**: 2125-2144.

Guede, I., Ortega, L.A., Zuluaga, M.C., Alonso-Olazabal, A., Murelaga, X., Pina, M., Gutierrez, F.J., Iacumin, P. (2017). Isotope analyses to explore diet and mobility in a medieval Muslim population at Tauste (NE Spain). *PLoS ONE* **12**: e0176572.

Guede, I., Ortega, L.A., Zuluaga, M.C., Alonso-Olazabal, A., Murelaga, X., Solaun, J.L., Sanchez, I., Azkarate, A. (2018). Isotopic evidence for the reconstruction of diet and mobility during village formation in the Early Middle Ages: Las Gobas (Burgos, northern Spain). *Archaeological and Anthropological Science* **10**: 2047-2058.

Guede, I., Zuluaga, M.C., Ortega, L.A., Alonso-Olazabal, A., Murelaga, X., Garcia Camino, I., Iacumin, P. (2020). Social structuration in medieval rural society based on stable isotope analysis of dietary habits and mobility patterns: San Juan de Momoitio (Biscay, north Iberian Peninsula). *Journal of Archaeological Science: Reports* **31**: 102300. DOI: 10.1016/j.jasrep.2020.102300.

Gugora, A., Dupras, T.L., Fóthi, E. (2018). Pre-dating paprika: Reconstructing childhood and adulthood diet at medieval (13th century CE) Solt-Tételhegy, Hungary from stable carbon and nitrogen isotope analyses. *Journal of Archaeological Science: Reports* **18**: 151-160.

Häberle, S., Fuller, B.T., Nehlich, O., Van Neer, W., Schibler, J., Hüster, Plogmann, H. (2016). Inter- and intraspecies variability in stable isotope ratio values of archaeological freshwater fish remains from Switzerland (11th–19th centuries AD). *Environmental Archaeology* **21**: 119-132.

Hakenbeck, S., Evans, J., Chapman, H., Fóthi, E. (2017). Practising pastoralism in an agricultural environment: An isotopic analysis of the impact of the Hunnic incursions on Pannonian populations. *PLoS ONE* **12**: e0173079. DOI: 10.1371/journal.pone.0173079.

Hakenbeck, S., McManus, E., Geisler, H., Grupe, G., O’Connell, T. (2010). Diet and Mobility in Early Medieval Bavaria: A Study of Carbon and Nitrogen Stable Isotopes. *American Journal of Physical Anthropology* **143**: 235-249.

Halffman, C.R., Velemínský, P. (2015). Stable isotope evidence for diet in early medieval Great Moravia (Czech Republic). *Journal of Archaeological Science: Reports* **2**: 1-8.

Halldórsdóttir, H.H., Rogers, B., DiRenno, F., Müldner, G., Gröcke, D.R., Barnicle, E., Chidimuro, B., Evans, M., Morley, R., Neff, M., Sharp, C., Simpson, A., Boucher, A. Montgomery, J. (2019). Continuity and individuality in Medieval Hereford, England: A stable isotope approach to bulk bone and incremental dentine. *Journal of Archaeological Science: Reports* **23**: 800-809.

Halley, D.J., Rosvold, J. (2014). Stable isotope analysis and variation in medieval domestic pig husbandry practices in northwest Europe: absence of evidence for a purely herbivorous diet. *Journal of Archaeological Science* **49**: 1-5.

Hamerow, H., Boogard, A., Charles, M., Forster, E., Holmes, M., Mckerracher, M., Neil, S., Bronk Ramsey, C., Stroud, E., Thomas, R. (2020). An Integrated Bioarchaeological Approach to the Medieval ‘Agricultural Revolution’: A Case Study from Stafford, England, c. AD 800–1200. *European Journal of Archaeology* **23**: 585-609.

Hamilton, J., Thomas, R. (2012). Pannage, Pulses and Pigs: Isotopic and Zooarchaeological Evidence for Changing Pig Management Practices in Later Medieval England. *Medieval Archaeology* **56**: 234-259.

Hammond, C., O’Connor, T. (2013). Pig diet in medieval York: carbon and nitrogen stable isotopes. *Archaeological and Anthropological Science* **5**: 123-127.

Hamre, S.S., Daux, V. (2016). Stable oxygen isotope evidence for mobility in medieval and post-medieval Trondheim, Norway. *Journal of Archaeological Science: Reports* **8**: 416-425.

Hannah, E.L., McLaughlin, T.R., Keaveney, E.M., Hakenbeck, S.E. (2018). Anglo-Saxon diet in the Conversion period: A comparative isotopic study using carbon and nitrogen. *Journal of Archaeological Science: Reports* **19**: 24-34.

Hattun, I. (2015). *“What’s on the Menu?”: Diet in Medieval Holland A stable carbon and nitrogen isotope analysis of bone “collagen” from early medieval Blokhuizen and late medieval Alkmaar*. Unpublished MA dissertation: Leiden University.

Haydock, H., Clarke, L., Craig-Atkins, E., Howcroft, R., Buckberry, J. (2013). Weaning at Anglo-Saxon Raunds: Implications for Changing Breastfeeding Practice in Britain Over Two Millennia. *American Journal of Physical Anthropology* **151**: 604-612.

Hemer, K.A., Evans, J., Chenery, C.A., Lamb, A.L. (2013). Evidence of early medieval trade and migration between Wales and the Mediterranean Sea region. *Journal of Archaeological Science* **40**: 2352-2359.

Hemer, K.A., Evans, J., Chenery, C.A., Lamb, A.L. (2014). No Man is an island: evidence of pre-Viking Age migration to the Isle of Man. *Journal of Archaeological Science* **52**: 242-249.

Hemer, K.A., Lamb, A.L., Chenery, C.A., Evans, J.A. (2017). A multi-isotope investigation of diet and subsistence amongst island and mainland populations from early medieval western Britain. *American Journal of Physical Anthropology* **162**: 423-440.

Herold, M. (2008). *Sex Differences in Mortality in Lower Austria and Vienna in the Early Medieval Period: An Investigation and Evaluation of Possible Contributing Factors*. Unpublished PhD dissertation: Universität Wien.

Herrscher, E. (2001). Alimentation d’une population historique: Analyse des données isotopiques de la nécropole Saint-Laurent de Grenoble (XIIIe-XVe siècle, France). [*Comptes Rendus de l'Académie des Sciences - Series III - Sciences de la Vie*](https://www.sciencedirect.com/science/journal/07644469) **324**: 479-487.

Higham, T., Warren, R., Belinskij, B., Härke, H., Wood, R. (2010). Radiocarbon dating, stable isotope analysis, and diet-derived offsets in 14C ages from the Klin-Yar site, Russian North Caucasus. *Radiocarbon* **52**: 653-670.

Howcroft, R., Eriksson, G., Lidén, K. (2012). Conformity in Diversity? Isotopic Investigations of Infant Feeding Practices in Two Iron Age Populations from Southern Öland, Sweden. *American Journal of Physical Anthropology* **149**: 217-230.

Hughes, S.S., Millard, A.R., Chenery, C.A., Nowell, G., Graham Pearson, D. (2018). Isotopic analysis of burials from the early Anglo-Saxon cemetery at Eastbourne, Sussex, U.K. *Journal of Archaeological Science: Reports* **19**: 513-525.

Hughes, S.S., Millard, A.R., Lucy, S.J., Chenery, C.A., Evans, J.A., Nowell, G., Graham Pearson, D. (2014). Anglo-Saxon origins investigated by isotopic analysis of burials from Berinsfield, Oxfordshire, UK. *Journal of Archaeological Science* **42**: 81-92.

Iacumin, P., Galli, E., Cavalli, F., Cecere, L. (2014). C4-consumers in Southern Europe: The case of Friuli V.G. (NE-Italy) during Early and Central Middle Ages. *American Journal of Physical Anthropology* **154**: 561-574.

Iacumin, P., Nikolaev, V., Genoni, L., Ramigni, M., Ryskov, Y.G., Longinelli, A. (2004). Stable isotope analyses of mammal skeletal remains of Holocene age from European Russia: A way to trace dietary and environmental changes. *Geobios* **37**: 37-47.

Inskip, S., Carroll, G., Waters-Rist, A., López-Costas, O. (2019). Diet and food strategies in a southern al-Andalusian urban environment during Caliphal period, Écija, Sevilla. *Archaeological and Anthropological Sciences* **11**: 3857–3874.

Inskip, S.A., Taylor, G.M., Zakrzewki, S.R., Mays, S.A., Pike, A.W.G., Llewellyn, G., Williams, C.M., Lee, O.Y-C., Wu, H.H.T., Minnikin, D.E., Besra, G.S., Stewart, G.R. (2015). Osteological, Biomolecular and Geochemical Examination of an Early Anglo-Saxon Case of Lepromatous Leprosy. *PLoS ONE* **10**: e0124282. DOI: 10.1371/journal.pone.0124282.

Jaouen, K., Colleter, R., Pietrzak, A., Pons, M.L., Clavel, B., Telmon, N., Crubézy, É, Hublin, J.J., Richards, M.P. (2018). Tracing intensive fish and meat consumption using Zn isotope ratios: evidence from a historical Breton population (Rennes, France). [*Scientific Reports*](https://www.nature.com/srep) **8**: DOI:10.1038/s41598-018-23249-x.

Jarman, C.L. (2012). *Identities Home and Abroad: An isotopic study of Viking Age Norway and the British Isles*. Unpublished MA dissertation: University of Oslo.

Jarman, C.L., Biddle, M., Higham, T., Bronk Ramsey, C. (2018). The Viking Great Army in England: new dates from the Repton charnel. *Antiquity* **92**: 183-199.

Jílková, M., Kaupová, S., Černíková, A., Poláček, L., Brůžek, J. (2019). Early medieval diet in childhood and adulthood and its reflection in the dental health of a Central European population (Mikulčice, 9^th^ –10^th^ centuries, Czech Republic). *Archives of Oral Biology* **107**: 104526. DOI: 10.1016/j.archoralbio.2019.104526.

Jiménez-Brobeil, S.A., Charisi, D., Laffranchi, Z., Maroto Benavides, R.M., Delgado Huertas, A., Milella, M. (2021). Sex differences in diet and life conditions in a rural Medieval Islamic population from Spain (La Torrecilla, Granada): An isotopic and osteological approach to gender differentiation in al-Andalus. *American Journal of Physical Anthropology*. DOI: 10.1002/ajpa.24277

Jiménez-Brobeil, S.A., Laffranchi, Z., Maroto, R.M., López Sánchez, F.A., Delgado Huertas, A. (2016). How royals feasted in the court of Pedro I of Castile: A contribution of stable isotope study to medieval history. *Journal of Archaeological Science: Reports* **10**: 424-430.

Jiménez-Brobeil, S.A., Maroto, R.M., Laffranchi, Z., Roca, M.G., Granados Torres, A., Delgado Huertas, A. (2020). Exploring diet in an isolated medieval rural community of Northern Iberia: The case study of San Baudelio de Berlanga (Soria, Spain). *Journal of Archaeological Science: Reports* **30**: 102218. DOI: 10.1016/j.jasrep.2020.102218.

Johansen, O.S., Gullisken, S., Nydal, R. (1986). δ^13^C and diet: Analysis of Norwegian human skeletons. *Radiocarbon* **28**: 754-761.

Jordana, X., Malgosa, A., Casté, B., Tornero, C. (2019). Lost in transition: the dietary shifts from Late Antiquity to the Early Middle Ages in the North Eastern Iberian Peninsula. *Archaeological and Anthropological Sciences* **11**: 3751–3763.

Jørkov, M.L.S., Heinemeier, J., Lynnerup, N. (2009). The Petrous Bone—A New Sampling Site for Identifying Early Dietary Patterns in Stable Isotopic Studies. *American Journal of Physical Anthropology* **138**: 199-20.

Kaal, J., López-Costas, O., Martínez Cortizas, A. (2016). Diagenetic effects on pyrolysis fingerprints of extracted collagen in archaeological human bones from NW Spain, as determined by pyrolysis-GC-MS. *Journal of Archaeological Science* **65**: 1-10.

Kancle, L., Montgomery, J., Gröcke, D.R., Caffell, A. (2018). From field to fish: Tracking changes in diet on entry to two medieval friaries in northern England. *Journal of Archaeological Science: Reports* **22**: 264-284.

Kaupová, S., Herrscher, E., Velemínský, P., Cabut, S., Poláček, L., Brůžek, J. (2014). Urban and rural infant-feeding practices and health in early medieval Central Europe (9th-10th Century, Czech Republic). *American Journal of Physical Anthropology* **155**: 635-651.

Kaupová, S., Schamall, D., Cvrček, J., Půtová, L., Velemínský, P., Teschler-Nicola, M. (2020). The dietary behavior of two early medieval individuals with temporomandibular ankyloses. *International Journal of Palaeopathology* **31**: 1-6.

Kaupová, S., Velemínský, P., Herrscher, E., Sládek, V., Macháček, J., Poláček, L., Brůžek, J. (2018). Diet in transitory society: isotopic analysis of medieval population of Central Europe (ninth–eleventh century AD, Czech Republic). *Archaeological and Anthropological Science* **10**: 923-942.

Kaupová, S., Velemínský, P., Stránská, P., Bravermanová, M., Frolíková, D. Tomková, K., Frolík, J. (2019). Dukes, elites, and commoners: dietary reconstruction of the early medieval population of Bohemia (9th–11th Century AD, Czech Republic). *Archaeological and Anthropological Science* **11**: 1887-1909.

Kendall, E.J., Millard, A., Beaumont, J., Gowland, R., Gorton, M., Gledhill, A. (2020). What Doesn’t Kill You: Early Life Health and Nutrition in Early Anglo-Saxon East Anglia. In Gowland, R., Halcrow, S. (eds)., *The Mother-Infant Nexus in Anthropology*. Cham. 103-123.

Kendall, E.J., Montgomery, J., Evans, J.A., Stantis, C., Mueller, V. (2013). Mobility, Mortality, and the Middle Ages: Identification of Migrant Individuals in a 14th Century Black Death Cemetery Population. *American Journal of Physical Anthropology* **150**: 210-222.

King, M. (2001). *A****nalysis of Diet in Byzantine Jordan: Isotopic Evidence in Human Dentine (Contribution to the Bioarchaeology of the Levant)*. Unpublished MA dissertation: University of Arkansas.**

Kline, S.A. (2015). *From Valley to Coast: An Isotopic Study of Diet in Southern Albania Across Three Millennia*. Unpublished MA dissetation: California State University.

Knipper, C., Held, P., Fecher, M., Nicklisch, N., Meyer, C., Schreiber, H., Zich, B., Metzner-Nebelsick, C., Hubensack, V., Hansen, L., Nieveler, E., Alt, K.W. (2015). Superior in Life-Superior in Death. Dietary Distinction of Central European Prehistoric and Medieval Elites. *Current Anthropology* **56**: 579-589.

Knipper, C., Maurer, A.F., Peters, D., Meyer, C., Brauns, M., Galer, S.J.G., von Freeden, U., Schöne, B., Meller, H., Alt, K.W. (2012). Mobility in Thuringia or mobile Thuringians: A strontium isotope study from early medieval Central Germany. In Kaiser, E., Burger, J., Schier, W. (eds.), *Population dynamics in prehistory and early history. New approaches using stable isotopes and genetic.* Boston. 287-310.

Knipper, C., Peters, D., Meyer, C., Maurer, A.F., Muhl, A., Schöne, B.R., Alt, K.W (2013). Dietary reconstruction in Migration Period Central Germany: a carbon and nitrogen isotope study. *Archaeological and Anthropological Sciences* **5**: 17-35.

Knudson, K.J., O’Donnabhain, B., Carver, C., Cleland, R., Price, T.D. (2012). Migration and Viking Dublin: paleomobility and paleodiet through isotopic analyses. *Journal of Archaeological Science* **39**: 308-320.

Kootker, L.M., Altena, E. (2012). *Bioarcheologisch onderzoek aan een kinderskelet uit Oegstgeest, plangebied Nieuw Rhijngeest-Zuid – SL Plaza*. IGBA-rapport 2011-07.

Kootker, L.M., van Lanen, R.J., Groenewoundt, B.J., Altena, E., Panhuysen, R.G.A.M., Jansma, E., Kars, H., Davies, G.R. (2019). Beyond isolation: understanding past human-population variability in the Dutch town of Oldenzaal through the origin of its inhabitants and its infrastructural connections. *Archaeological and Anthropological Sciences* **11**: 755-775.

Kosiba, S.B., Tykot, R.H., Carlsson, D. (2007). Stable isotopes as indicators of change in the food procurement and food preference of Viking Age and Early Christian populations on Gotland (Sweden). *Journal of Anthropological Archaeology* **26**: 394-411.

Kovačiková, L., Kaupová, S., Poláček, L., Velemínský, P., Limburský, P., Brůžek, J. (2020). Pig-Breeding Management in the Early Medieval Stronghold at Mikulčice (Eighth–Ninth Centuries, Czech Republic). *Environmental Archaeology*. DOI: 10.1080/10.1080/14614103.2020.1782583.

Kovačiková. L., Trojánková, O., Starec, P., Meduna, P., Limburský, P. (2020). Livestock as an indicator of socioeconomic changes in Medieval Prague (Czech Republic). *Archaeological and Anthropological Science* **12**: 283. DOI: 10.1007/s12520-020-01229-5

Krylasova, N.B., Brykhova, N.G., Burova, N.D. (2016). The Role of Nutrition in the Biological Adaptation of the Medieval Population of the Cis-Ural Perm Region (Archeological and Anthropological evidence). *International Journal of Environmental & Science Education* **11**: 4812-4824.

Krzewińska, M., Kjellström, A., Günther, T., Hedenstierna-Jonson, C., Zachrisson, T., Omrak, A., Yaka, R., Kılınç, G.M., Somel, M., Sobrado, V., Evans, J., Knipper, C., Jakobsson, M., Storå, J., Götherström, A. (2018). Genomic and Strontium Isotope Variation Reveal Immigration Patterns in a Viking Age Town. *Current Biology* **28**: 2730-2738.

Kwok, C.S., Garvie-Lok, S., Katzenberg, M.A. (2018). Exploring variation in infant feeding practices in Byzantine Greece using stable isotope analysis of dentin serial sections. *International Journal of Osteoarchaeology* **28**: 563-578.

Laffranchi, Z., Mazzucchi, A., Thompson, S., Delgado-Hueltas, A., Granados-Torres, A., Milella, M. (2020). Funerary reuse of a Roman amphitheatre: Palaeodietary and osteological study of Early Middle Ages burials (8^th^ and 9^th^ centuries AD) discovered in the Arena of Verona (Northeastern Italy). *International Journal of Osteoarchaeology* **30**: 435-448.

Lahtinen, M. (2017). Isotopic evidence for environmental adaptation in Medieval Iin Hamina, Northern Finland. *Radiocarbon* **59**: 1117-1131.

Lahtinen, M., Arppe, L., Nowell, G. (2021). Source of strontium in archaeological mobility studies – marine diet contribution to the isotopic composition. *Archaeological and Anthropological Sciences* **13**. DOI: 10.1007/s12520-020-01240-w

Lahtinen, M., Salmi, A.K. (2019). Mixed Livelihood Society in Iin Hamina – a Case Study of Medieval Diet in the Northern Ostrobothnia, Finland. *The Journal of Human Palaeoecology* **24**: 1-14.

Lamb, A., Evans, J.E., Buckley, R., Appleby, J. (2014). Multi-isotope analysis demonstrates significant lifestyle changes in King Richard III. *Journal of Archaeological Science* **50**: 559-565.

Lamb, A., Melikian, M., Ives, R., Evans, J. (2012). Multi-isotope analysis of the population of the lost medieval village of Auldhame, East Lothian, Scotland. *Journal of Analytical Atomic Spectrometry* **27**: 765-777.

Ledogar, S.H., Karsten, J.K., Madden, G.D., Schmidt, R., Sokohatskyi, M.P., Feranec, R.S. (2018). New AMS dates for Verteba Cave and stable isotope evidence of human diet in the Holocene forest-steppe Ukraine. *Radiocarbon* **61**: 141-158.

Leslie, B.G. (2012). *Residential Mobility in the Rural Greek Past: A Strontium Isotope Investigation*. Unpublished MA dissertation: University of Alberta.

Lidén, K., Nelson, E.D. (1994). Stable carbon isotopes as dietary indicator, in the Baltic area. *Fornvännen* **89**: 13-21.

Lightfoot, E., Naum, M., Kadakas, V., Russow, E. (2016). The influence of social status and ethnicity on diet in Mediaeval Tallinn as seen through stable isotope analysis. *Estonian Journal of Archaeology* **20**: 81-107.

Lightfoot, E., O’Connell, T., Stevens, R.E., Hamilton, J., Hey, G., Hedges, R.E.M. (2009). An investigation into diet at the site of Yarnton, Oxfordshire, using stable carbon and nitrogen isotopes. *Oxford Journal of Archaeology* **28**: 301-322.

Lightfoot, E., Pomeroy, E., Grant, J., O’Connell, T.C., le Roux, P., Zakrzewski, S., Inskip, S., Benady, S., Finlayson, C., Finlayson, G., Lane, K. (2020). Sea, sickness and cautionary tales: a multi-isotope study from a post-mediaeval hospital at the city-port of Gibraltar (AD 1462–1704). *Archaeological and Anthropological Sciences* **12**: 273. DOI: 10.1007/s12520-020-01220-0

Lightfoot, E., Šlaus, M., O’Connell, T.C. (2012). Changing Cultures, Changing Cuisines: Cultural Transitions and Dietary Change in Iron Age, Roman, and Early Medieval Croatia. *American Journal of Physical Anthropology* **148**: 543-556.

Linderholm, A., Andersson, K., Mörth, C.M., Grundberg, L., Hårding, B., Lidén, K. (2008). An early Christian cemetery at Bjorned in northern Sweden. Stable isotope analyses of skeletal material. *Journal of Swedish Antiquarian Research* **103**: 176-189.

Linderholm, A., Hedenstierna-Jonson, C., Svenks, O., Lidén, K. (2008). Diet and status in Birka: stable isotopes and grave goods compared. *Antiquity* **82**: 446-461.

Linderholm, A., Kjellström, A. (2011). Stable isotope analysis of a medieval skeletal sample indicative of systemic disease from Sigtuna Sweden. *Journal of Archaeological Science* **38**: 925-933.

Lisowska-Gaczorek, A., Kozieł, S., Cienkosz-Stepánczak, B., Mądrzyk, K., Pawlyta, J., Gronkiewicz, S., Wołoszyn, M., Szostek, K. (2016). An analysis of the origin of an early medieval group of individuals from Gródek based on the analysis of stable oxygen isotopes. *Journal of Comparative Human Biology* **67**: 313-327.

López-Aceves, J.M. (2019). *Diet and dynamic of the last Muslims in Algarve during the 12th-13th AD*. Unpublished MA dissertation: University of Évora.

López-Costas, O., Müldner, G. (2016). Fringes of the empire: Diet and cultural change at the Roman to post-Roman transition in NW Iberia. *American Journal of Physical Anthropology* **161**: 141-154.

López-Costas, O., Müldner, G. (2019). Boom and bust at a medieval fishing port: dietary preferences of fishers and artisan families from Pontevedra (Galicia, NWSpain) during the Late Medieval and Early Modern Period. *Archaeological and Anthropological Sciences* **11**: 3717–3731.

Lubritto, C., García-Collado, M.I., Ricci, P., Altieri, S., Sirignano, C., Quirós Castillo, J.A. (2017). New Dietary Evidence on Medieval Rural Communities of the Basque Country (Spain) and Its Surroundings from Carbon and Nitrogen Stable Isotope Analyses: Social Insights, Diachronic Changes and Geographic Comparison. *International Journal of Osteoarchaeology* **27**: 984-1002.

Lubritto, C., Sirignano, C., Ricci, P., Passariello, I., Quiros Castillo, J.A. (2013). Radiocarbon Chronology and Paleodiet studies on the Medieval rural site of Zaballa (Spain): Preliminary insights into the social archaeology of the site. *Radiocarbon* **55**: 1222-1232.

Lucy, S., Newman, R., Dodwell, N., Hills, C., Dekker, M., O’Connell, T., Riddler, I., Walton Rogers, P. (2009). The Burial of a Princess? The Later Seventh-Century Cemetery at Westfield Farm, Ely. *The Antiquaries Journal* **89**: 81-141.

Luxton, S.A. (2015). *Exploring the relationship between diet and osteoporosis in Medieval Portugal using stable isotope analysis*. Unpublished MA dissertation: University of Alaska Fairbanks.

Ma, Y., Bockmann, R., Stevens, S.T., Roudesli-Chebbi, S., Amaro, A., Brozou, A., Fuller, B.T., Mannino, M.A. (2021). Isotopic reconstruction of diet at the Vandalic period (c. 5th – 6th centuries AD) Theodosian Wall cemetery at Carthage, Tunisia. *International Journal of Osteoarchaeology*. DOI: 10.1002/oa.2958

MacKinnon, A.T., Passalacqua, N.V., Bartelink, E.J. (2019). Exploring diet and status in the Medieval and Modern periods of Asturias, Spain, using stable isotopes from bone collagen. *Archaeological and Anthropological Sciences* **11**: 3837–3855.

MacPherson, P.M. (2005). *Tracing Change: An Isotopic Investigation of Anglo-Saxon Childhood Diet*. Unpublished PhD dissertation. University of Sheffield.

MacRoberts, R.A., Barrocas Dias, C.M., Fernandes, T.M., Santos, A.L., Umbelino, C., Gonçalves, A., Santos, J., Ribeiro, S., Schöne, B.R., Barros, F., Correia, F., Vasconcelos Vilar, H., Maurer, A.F. (2020). Diet and mobility during the Christian conquest of Iberia: The multi-isotopic investigation of a 12th–13th century military order in Evora, Portugal. *Journal of Archaeological Science: Reports* **30**: 102210. DOI: 10.1016/j.jasrep.2020.102210.

Magalhães Ribeiro, C. (2019). *Nas Cozinhas Medievais de Estremoz. Estudo paleobiológico e análise de isótopos estáveis de uma amostra osteológica da população inumada no Rossio Marquês de Pombal, Estremoz.* Unpublished master dissertation: Universidade de Coimbra.

Marinato, M. (2014). *Analisi isotopiche e bioarcheologia come fonti per lo studio del popolamento tra tardo antico e alto medioevo in Italia settentrionale. Dati a confronto per le province di Bergamo, Modena e Verona*. Unpublished PhD dissertation: Università degli studi di Padova.

Marinato, M. (2017). Analisi degli isotopi stabili delle sepolture altomedievali. In Chavarría Arnau, A. (ed.), *Ricerche sul centro episcopale di Padova. Scavi 2011-2012*. Mantova. 151-154.

Marinato, M. (2018). Potenzialità di un approccio multidisciplinare per lo studio del popolamento antico: il territorio di Bergamo tra tarda antichità e alto medioevo. In Giostra, C. (ed.), *Città e campagna: culture, insediamenti, economia (secc. VI-IX)*. ‘II Incontro per l’Archeologia barbarica Milano, 15 maggio 2017’. Mantova. 75-96.

Marshall, P., van der Plicht, J., Cook, G.T., Grootes, P.M., Beavan-Athfield, N., Buzinny, M. (2010). Scientific dating evidence. In Thomas, G. (ed.), *The Later Anglo-Saxon Settlement at Bishopstone: A Download Manor in the Making*. York. 197-206.

Martínez‐Jarreta, B., Sosa, C., Laliena, C., Budowle, B., Hedges, R.E.M. (2018). Stable Isotope and Radiocarbon Dating of the Remains of the Medieval Royal House of Aragon (Spain) Shed Light on Their Diets, Life Histories and Identities. *Archaeometry* **60**: 366-382.

Maxwell, A. (2019). *Exploring Variations in Diet and Migration from Late Antiquity to the Early Medieval Period in the Veneto, Italy: A Biochemical Analysis*. Unpublished PhD dissertation: University of South Florida.

Mays, S. (1997). Carbon Stable Isotope Ratios in Mediaeval and Later Human Skeletons from Northern England. *Journal of Archaeological Science* **24**: 561-567.

Mays, S., Beavan, N. (2012). An investigation of diet in early Anglo-Saxon England using carbon and nitrogen stable isotope analysis of human bone collagen. *Journal of Archaeological* Science **39**: 867-874.

Mays, S., Fryer, R., Pike, A.W.G., Cooper, M.J., Marshall, P. (2017). A multidisciplinary study of a burnt and mutilated assemblage of human remains from a deserted Mediaeval village in England. *Journal of Archaeological Science: Reports* **16**: 441-455.

McConnan-Borstad, C., Garvie-Lok, S., Katsonopoulou, D. (2018). Diet at ancient Helike, Achaea, Greece based on stable isotope analysis: From the Hellenistic to the Roman and Byzantine periods. *Journal of Archaological Science: Reports* **18**: 1-10.

McGlynn, G. (2007). *Using 13C-, 15N- and 18O stable isotope analysis of human bone tissue to identify transhumance, high altitude habitation and reconstruct palaeodiet for the early medieval Alpine population at Volders,* Austria. Unpublished PhD dissertation: Ludwig-Maximilians-Universität München.

McGovern, T.H., Vésteinsson, O., Friðriksson, A., Church, M., Lawson, I., Simpson, I.A., Einarsson, A., Dugmore, A., Cook, G., Perdikaris, S., Edwards, K.J., Thomson, A.M., Adderley, W.P., Newton, A., Lucas, G., Edvardsson, R., Aldred, O., Dunbar, E. (2007). Landscapes of Settlement in Northern Iceland: Historical Ecology of Human Impact and Climate Fluctuation on the Millennial Scale. *American Anthropologist* **109**: 27-51.

McKenzie, C.J., Murphy, E.M., Guiry, E, Donnelly, C.J., Beglane, F. (2020). Diet in Medieval Gaelic Ireland: A multiproxy study of the human remains from Ballyhanna, Co. Donegal. *Journal of Archaeological Science* **121**: 105203. DOI: 10.1016/j.jas.2020.105203

McManus, E., Montgomery, J., Evans, J., Lamb, A., Brettell, R., Jelsma, J. (2013). “To the Land or to the Sea”: Diet and Mobility in Early Medieval Frisia. *Journal of Island and Coastal Archaeology* **8**: 255-277.

Meijer, J., Dolphin, A.E., Yakymchuk, C., Gervers, M. (2019). Interpreting medieval mobility from burials at the rock-hewn church of St. Georges, Gurat (France): Insights from strontium isotope analysis of bones and teeth. *International Journal of Osteoarchaeology* **29**: 574-583.

Miclon, V., Gaultier, M., Genies, C., Cotté, O., Yvernault, F., Herrscher, E. (2019). Social Characterization of the Medieval and Modern Population from Joué-lès-Tours (France): Contribution of Oral Health and Diet. *Bulletins et mémoires de la Société d'anthropologie de Paris* **31**: 77-92.

Millard, A.R., Jimenez-Cano, N.G., Lebrasseur, O., Sakai, Y. (2013). Isotopic Investigation of Animal Husbandry in the Welsh and English Periods at Dryslwyn Castle, Carmarthenshire, Wales. *International Journal of Osteoarchaeology* **23**: 640-650.

Mion, L., Herrscher, E., André, G., Hernandez, J., Donat, R., Fabre, M., Forest, V., Salazar-García, D.C. (2019). The influence of religious identity and socio-economic status on diet over time, an example from medieval France. *Archaeological and Anthropological Sciences* **11**: 3309-3327.

Mion, L., Herrscher, E., Hernandez, J., Donat, R., Tarrou, L., Fabre, M., Forest, V. (2017). Mauguio (Hérault). L’alimentation dans le sud-est de la France au haut Moyen Age. Le cas des sujets de Lallemand à Mauguio: un exemple d’application de l’outil isotopique. *Archéologie du Midi Médiéval* **35**: 300-311.

Miszkiewicz, J.J., Stewart, T.J., Deter, C.A., Fahy, G.E., Mahoney, P. (2019). Skeletal Health in Medieval Societies: Insights from Ancient Bone Collagen Stable Isotopes and Dental Histology. In Miszkiewicz J., Brennan-Olsen S., Riancho J. (eds), *Bone Health*. Singapore. 17-34.

Mitchell, P.D., Millard, A.R. (2009). Migration to the Medieval Middle East with the Crusades. *American Journal of Physical Anthropology* **140**: 518-525.

Moles, A. (2012). *A Stable Isotope Analysis Study for Dietary Reconstruction at the Multi-Period Site of Mesembria on the Black Sea*. Unpublished MA dissertation: University of Edinburgh.

Montgomery, J., Evans, J.A., Chenery, C.A., Müldner, G. (2009). Stable isotope analysis of bone. In Carver, M., Hills, C., Scheschkewitz, J. (eds.), *Wasperton: A Roman, British and Anglo-Saxon Community in Central England*. Woodbridge. 48-49.

Montgomery, J., Evans, J.A., Neighbour, T. (2003). Sr isotope evidence for population movement within the Hebridean Norse community of NW Scotland. *Journal of the Geological Society* **160**: 649-653.

Montgomery, J., Grimes, V., Buckberry, J., Evans, J.A., Richards, M.P., Barrett, J.H. (2014). Finding Vikings with Isotope Analysis: The View from Wet and Windy Islands. *Journal of the North Atlantic* **7**: 54-70.

Moore, J., Hamilton, D. Speed, G. (2019). Scientific analyses. In Speed, G., Holst, M. (ed.). *A1 Leeming to Barton. Death, Burial and Identity. 3000 Years of Death in the Vale of* Mowbray. Marwood House. 579-599.

Müldner, G., Britton, K., Ervynck, A. (2014). Inferring animal husbandry strategies in coastal zones through stable isotope analysis: new evidence from the Flemish coastal plain (Belgium, 1ste15th century AD). *Journal of Archaeological Science* **41**: 322-332.

Müldner, G., Montgomery, J., Cook, G., Ellam, R., Gledhill, A., Lowe, C. (2009). Isotopes and individuals: diet and mobility among the medieval Bishops of Whithorn. *Antiquity* **83**: 1119-1133.

Müldner, G., Richards, M.P. (2005). Fast or feast: reconstructing diet in later medieval England by stable isotope analysis. *Journal of Archaeological Science* **32**: 39-48.

Müldner, G., Richards, M.P. (2007a). Diet and Diversity at Later Medieval Fishergate: The Isotopic Evidence. *American Journal of Physical Anthropology* **134**: 162-174.

Müldner, G., Richards, M.P. (2007b). Stable Isotope Evidence for 1500 Years of Human Diet at the City of York, UK. *American Journal of Physical Anthropology* **133**: 682-697.

Mulville, J., Madgwick, R., Stevens, R., O’Connell, T., Craig, O., Powell, A., Sharples, N., Pearson, M.P. (2009). Isotopic Analysis of Faunal Material from South Uist, Western Isles, Scotland. *Journal of the North Atlantic* **2**: 51-59.

Naumann, E., Glørstad, A.Z.T., Breiby, M.P., Mills, R.D., Fullagar, P.D. (2019). Who were the first urban settlers of Oslo? A discussion of Early Medieval urbanization based on isotopic analyses of human remains. *Archaeometry* **61**: 1111-1128.

Naumann, E., Krzewińska, M., Götherström, A., Eriksson, G. (2014). Slaves as burial gifts in Viking Age Norway? Evidence from stable isotope and ancient DNA analyses. *Journal of Archaeological Science* **41**: 533-540.

Naumann, E., Price, T.D., Richards, M.P. (2014). Changes in Dietary Practices and Social Organization During the Pivotal Late Iron Age Period in Norway (AD 550–1030): Isotope Analyses of Merovingian and Viking Age Human Remains. *American Journal of Physical Anthropology* **155**: 322-331.

Nehlich, O., Barrett, J.H., Richards, M.P. (2013). Spatial variability in sulphur isotope values of archaeological and modern cod (Gadus morhua). *Rapid Communications in Mass Spectrometry* **27**: 2255-2262.

Nehlich, O. Fuller, B.T., Márquez-Grant, N., Richards, M.P. (2012). Investigation of Diachronic Dietary Patterns on the Islands of Ibiza and Formentera, Spain: Evidence from Sulfur Stable Isotope Ratio Analysis. *American Journal of Physical Anthropology* **149**: 115-124.

Nelson, D.E., Heinemeier, J., Lynnerup, N., Sveinbjörnsdóttir, Á., Arneborg, J. (2012). An Isotopic Analysis of the Diet of the Greenland Norse. *Journal of the North Atlantic* **3**: 93-118.

Nelson, D.E., Heinemeier, J., Møhl, J., Arneborg, J. (2012). Isotopic Analyses of The Domestic Animals of Norse Greenland. *Journal of North Atlantic* **3**: 77-92.

Nitsch, E.K. (2012). *Stable isotope evidence for diet change in Roman and Medieval Italy: local, regional and continental perspectives*. Unpublished PhD Dissertation: University of Oxford.

Noche-Dowdy, L.D. (2015). *Multi-Isotope Analysis to Reconstruct Dietary and Migration Patterns of an Avar Population from Sajópetri, Hungary, AD 568-895*. Unpublished MA dissertation: University of Florida.

Novak, M., Howcroft, R., Pinhasi, R. (2017). Child Health in Five Early Medieval Irish Sites: A Multidisciplinary Approach. *International Journal of Osteoarchaeology* **27**: 398-408.

Novak, M., Howcroft, R., Pinhasi, R., Šlaus, M. (2016). Dietary trends in early medieval Croatia as evidenced by stable isotope analysis. *American Journal of Physical Anthropology* **159**: 242.

Núñez, M., Äikäs, T., Aspi, J., Eriksson, G., Heino, M., Lidén, K., Oinonen, M., Okkonen, J., Salmi, A.K. (2021). Animal remains from Saami offering places: Glimpses of human-animal relations from Finnish Lapland AD 1000-1900. *Monographs of the Archaeological Society of Finland* **9**: 61-78.

O’Connell, T., Ballantyne, R.M., Hamilton-Dyer, S., Margaritis, E., Oxford, S., Pantano, W., Millett, M., Keay, S.J. (2019). Living and dying at the *Portus Romae*. *Antiquity* **93**: 719-734.

O’Connell, T., Lawler, A. (2009). Stable isotope analysis of human and faunal remains. In Lucy, S., Tipper, J., Dickens, A. (eds.), *The Anglo-Saxon Settlement and Cemetery at Bloodmoor Hill, Carlton Colville, Suffolk*. Cambridge. 317-321.

O’Connell, T., Wilson, E.J. (2008). Stable isotope analysis of human remains from the Anglo-Saxon cemetery at Butler’s Field, Lechdale, Gloucestershire: dietary and social implications. In Boyle, A., Jennings, D., Miles, D., Palmer, S. (eds.), *The Anglo-Saxon Cemetery at Butler's Field, Lechdale, Gloucestershire*. Oxford.

Oinonen, M., Alenius, T., Arppe, L., Bocherens, H., Etu-Sihvola, H., Helama, S., Huhtamaa, H., Lahtinen, M., Mannermaa, K., Onkamo, P., Palo, J., Sahantila, A., Salo, K., Sundell, T., Vanhanen, S., Wessman, A. (2020). Buried in water, burdened by nature — Resilience carried the Iron Age people through Fimbulvinter. *PLoS ONE* **15**: e0231787. DOI: 10.1371/journal.pone.0231787

Olsen, J., Dahlström, H., Poulsen, B. (2019). The chronology of Medieval Copenhagen. *Radiocarbon* **61**: 1675-1683.

Olsen, K.C., White, C.D., Longstaffe, F.J., Rühli, F.J., Warinner, C., Salazar-García, D.C. (2018). Isotopic anthropology of rural German medieval diet: intra and inter-population variability. *Archaeological and Anthropological Sciences* **10**: 1053–1065.

Olsen, K.C., White, C.D., Longstaffe, F.J., Von Heyking, K., McGlynn, G., Grupe, G., Rühli, F.J. (2014). Intraskeletal Isotopic Compositions (d13C, d15N) of Bone Collagen: Nonpathological and Pathological Variation. *American Journal of Physical Anthropology* **153**: 598-604.

Oras, E., Tõrv, M., Jonunks, T., Malve, M., Radini, A., Isaksson, S., Gledhill, A., Kekišev, O., Vahur, S., Leito, I. (2018). Social food here and hereafter: Multiproxy analysis of gender-specific food consumption in conversion period inhumation cemetery at Kukruse, NE-Estonia. *Journal of Archaeological Science* **97**: 90-101.

Ortega-González, A.F. (2019). *Diet and dynamic of the first Christians in Algarve during the 13th-14th AD*. Unpublished MA dissertation: University of Évora.

Ortega, L.A., Guede, I., Zuluaga, M.C., Alonso-Olazabal, A., Murelaga, X., Niso, J., Loza, M., Quirós Castillo, J.A. (2013). Strontium isotopes of human remains from the San Martín de Dulantzi graveyard (Alegría-Dulantzi, Álava) and population mobility in the Early Middle Ages. *Quaternary International* **303**: 54-63.

Orton, D.C., Makowiecki, D., De Roo, T., Johnstone, C., Harland, J., Jonsson, L., Heinrich, D., Enghoff, I.B, Lõugas, L., Van Neer, W., Ervynck, A., Hufthammer, A.K., Amundsen, C., Jones, A.K.G., Locker, A., Hamilton-Dyer, S., Pope, P., MacKenzie, B.R., Richards, M.P., O’Connell, T., Barrett, J.H. (2011). Stable Isotope Evidence for Late Medieval (14th–15th C) Origins of the Eastern Baltic Cod (Gadus morhua) Fishery. *PLoS ONE* **6**: e27568. DOI: 10.1371/journal.pone.0027568.

Otten, T., Evans, J., Lamb, A., Müldner, G., Pirson, A., Teegen, W.R. (2011). Ein Frühbyzantinisches Waffengrab aus Pergamon. Interpretationsmöglichkeiten aus archäologischer und naturwissenschaftlicher Sicht. *Istanbuler Mitteilungen* **61**: 347-422.

Page, K. (2014). *Bioarchaeological assessment of diet and changes in femoral and humeral stable isotopic values among subadults at Medieval Alytus, Lithuania*. Unpublished MA dissertation: University of Central Florida.

Paladin, A., Moghaddam, N., Stawinoga, A.E., Siebke, I., Depellegrin, V., Tecchiati, U., Lösch, S., Zink, A. (2020). Early medieval Italian Alps: reconstructing diet and mobility in the valleys. *Archaeological and Anthropological Science* **12**. DOI: 10.1007/s12520-019-00982-6.

Palincaş, N. Simion, C.A., Sava, G.O., Gâza, O., Sava, T.B., Constantinescu, B., Stan, D., Manea, M.M. (2019). Archaeometry and Individual Biographies: Evidence from Radiocarbon Dating, Isotope-Based Diet Reconstruction and Metal Composition from the 14th-17th-Century Cemetery in Bărăşti (Southern Romania). In Palincaş, N., Ponta, C.C. (eds.), *Bridging Science and Heritage in the Balkans. Studies in archaeometry, cultural heritage restoration and conservation*. Oxford. 16-28.

Pennycook, C. (2008). *A Stable Isotope Reconstruction of Byzantine and Frankish Greek Diet in the Valley of Stymphalos*. Unpublished MA dissertation: University of Alberta.

Peschel, E.M., Carlsson, D., Bethard, J., Beaudry, M.C. (2017). Who resided in Ridanäs?: A study of mobility on a Viking Age trading port in Gotland, Sweden. *Journal of Archaeological Science: Reports* **13**: 175-184.

Pescucci, L., Battistini, A., De Angelis, F., Catalano, P. (2013). Vivere al centro di Roma nell’VIII secolo D.C. Indicazioni Antropologiche. *Bollettino di Archeologia On Line* **4**: 113-138.

Petersone-Gordina, E., Montgomery, J., Millard, A.R., Roberts, C., Gröcke, D., Gerhards, G. (2020). Investigating the dietary life histories and mobility of children buried in St Gertrude Church cemetery, Riga, Latvia, 15th–17th centuries AD. *Archaeometry*. DOI: 10.1111/arcm.12520.

Petersone-Gordine, E., Roberts, C., Millard, A.R., Montgomery, J., Gerhards, G. (2018). Dental disease and dietary isotopes of individuals from St Gertrude Church cemetery, Riga, Latvia. *PLoS ONE* **13**: e0191757. DOI: 10.1371%2Fjournal.pone.0191757.

Pickard, C., Girdwood, L.K., Kranioti, E., Márquez-Grant, N., Richards, M.P., Fuller, B.T. (2017). Isotopic evidence for dietary diversity at the mediaeval Islamic necropolis of Can Fonoll (10th to 13th centuries CE), Ibiza, Spain. *Journal of Archaeological Science: Reports* **13**: 1-10.

Pitts, M., Bayliss, A., McKinley, J., Bylston, A., Budd, P., Evans, J., Chenery, C., Reynolds, A., Semple, S.J. (2002). An Anglo-Saxon decapitation and burial at Stonehenge. *Wiltshire archaeological and Natural History Magazine* **95**: 131-146.

Plecerová, A., Kaupová-Drtikolová, S., Šmerda, J., Stloukal, M. (2020). Dietary reconstruction of the Moravian Lombard population (Kyjov, 5^th^–6^th^ centuries AD, Czech Republic) through stable isotope analysis (δ13C, δ15N). *Journal of Archaeological Science: Reports* **29**: 102062. DOI: 10.1016/j.jasrep.2019.102062.

Polet, C., Katzenberg, M.A. (2003). Reconstruction of the diet in a mediaeval monastic community from the coast of Belgium. *Journal of Archaeological Science* **30**: 525-533.

Pollard, A.M., Ditchfield, P., Piva, E., Wallis, S., Falys, C., Ford, S. (2012). ‘Sprouting like cockle amongst the wheat’: The St. Brice’s day massacre and the isotopic analysis of human bones from St. John’s College, Oxford. *Oxford Journal of Archaeology* **31**: 83-102.

Prevedorou, E., Díaz-Zorita Bonilla, M., Romero, A., Buikstra, J.E., de Miguel Ibáñez, M.P., Knudson, K.J. (2010). Residential Mobility and Dental Decoration in Early Medieval Spain: Results from the Eighth Century Site of Plaza del Castillo, Pamplona. *Dental Anthropology* **23**: 42-52.

Price, T.D. (2013). Human mobility at Uppåkra. A preliminary report on isotopic proveniencing. In Hårdh, B., Larsson, L. (Eds.), *Studies at Uppåkra, An Iron Age City in Scania, Sweden*. Lund. 157–169.

Price, T.D., Arcini, C., Gustin, I., Drenzel, L., Kalmring, S. (2018). Isotopes and human burials at Viking Age Birka and the Mälaren region, east central Sweden. *Journal of Anthropological Archaeology* **49**: 19-38.

Price, T.D., Frei, K.M., Dobat, A.S., Lynnerup, N., Bennike, P. (2011). Who was in Harold Bluetooth's army? Strontium isotope investigation of the cemetery at the Viking Age fortress at Trelleborg, Denmark. *Antiquity* **85**: 476-489.

Price, T.D., Gestsdóttir, H. (2006). The first settlers of Iceland: an isotopic approach to colonisation. *Antiquity* **80**: 130-144.

Price, T.D., Moiseyev, V., Grigoreva, N. (2019). Vikings in Russia: origins of the medieval inhabitants of Staraya Ladoga. *Archaeological and Anthropological Science* **11**: 6093–6109.

Price, T.D., Naum, M., Bennike, P., Lynnerup, N., Frei, K.M., Wagnkilde, H., Pind, T., Nielsen, F.O. (2012). Isotopic investigation of human provenience at the eleventh century cemetery of Ndr. Grødbygård, Bornholm, Denmark. *Danish Journal of Archaeology* **1**: 93-112.

Price, T.D., Nielsen, J.N., Frei, K.M., Lynnerup, N. (2012). Sebbersund: isotopes and mobility in an 11^th^-12^th^ c. AD Danish churchyard. *Journal of Archaeological Science* **39**: 3714-3720.

Price, T.D., Peets, J., Allmäe, R., Maldre, L., Oras, E. (2016). Isotopic provenancing of the Salme ship burials in Pre-Viking Age Estonia. *Antiquity* **90**: 1022-1037.

Price, T.D., Peets, J., Allmäe, R., Maldre, L., Price, N. (2020). Human remains, context, and place of origin for the Salme, Estonia, boat burials. *Journal of Anthropological Archaeology* **58**: 101149. DOI: 10.1016/j.jaa.2020.101149.

Price, T.D., Prangsgaard, K., Kanstrup, M., Bennike, P., Frei, K.M. (2015). Galgedil: isotopic studies of a Viking cemetery on the Danish island of Funen, AD 800–1050. *Danish Journal of Archaeology* **2**: 129-144.

Privat, K.L., O’Connell, T., Richards, M.P. (2002). Stable Isotope Analysis of Human and Faunal Remains from the Anglo-Saxon Cemetery at Berinsfield, Oxfordshire: Dietary and Social Implications- *Journal of Archaeological Science* **29**: 779-790.

Reitsema, L.J., Crews, D.E., Polcyn, M. (2010). Preliminary evidence for medieval Polish diet from carbon and nitrogen stable isotopes. *Journal of Archaeological Science* **37**: 1413-1423.

Reitsema, L.J., Kozłowski, T., Crews, D.E., Katzenberg, M.A., Chudziak, W. (2017). Resilience and local dietary adaptation in rural Poland, 1000–1400 CE. *Journal of Anthropological Archaeology* **45**: 38-52.

Reitsema, L.J., Kozłowski, T., Makowiecki, D. (2013). Humane environment interactions in medieval Poland: a perspective from the analysis of faunal stable isotope ratios. *Journal of Archaeological Science* **40**: 3636-3646.

Reitsema, L.J., Vercellotti, G. (2012). Stable isotope evidence for sex- and status-based variations in diet and life history at Medieval Trino Vercellese, Italy. *American Journal of Physical Anthropology* **148**: 589-600.

Reitsema, L.J., Vercellotti, G., Boano, R. (2016). Subadult dietary variation at Trino Vercellese, Italy, and its relationship to adult diet and mortality. *American Journal of Physical Anthropology* **160**: 653-664.

Ricci, P., Mongelli, V., Vitiello, A., Campana, S., Sirignano, C., Rubino, M., Fornaciari, G., Lubritto, C. (2012). The privileged burial of the Pava Pieve (Siena, 8th Century AD). *Rapid Communications in Mass Spectometry* **26**: 2393-2398.

Riccomi, G., Minozzi, S., Zech, J., Cantini, F., Giuffra, V., Roberts, P. (2020). Stable isotopic reconstruction of dietary changes across Late Antiquity and the Middle Ages in Tuscany. *Journal of Archaeological Science: Reports* **33**: 102546. DOI: 10.1016/j.jasrep.2020.102546

Richards, M.P., Fuller, B.T., Molleson, T.I. (2006). Stable isotope palaeodietary study of humans and fauna from the multi-period (Iron Age, Viking and Late Medieval) site of Newark Bay, Orkney. *Journal of Archaeological Science* **33**: 122-131.

Richards, M.P., Mays, S., Fuller, B.T. (2002). Stable Carbon and Nitrogen Isotope Values of Bone and Teeth Reflect Weaning Age at the Medieval Wharram Percy Site, Yorkshire, UK. *American Journal of Physical Anthropology* **119**: 205-210.

Roberts, C.A., Millard, A.R., Nowell, G.M., Gröcke, D.R., Macpherson, C.G., Pearson, D.G., Evans, D.H. (2013). Isotopic Tracing of the Impact of Mobility on Infectious Disease: The Origin of People with Treponematosis Buried in Hull, England, in the Late Medieval Period. *American Journal of Physical Anthropology* **150**: 273-285.

Roffey, S., Tucker, K., Filipek-Ogden, K., Montgomery, J., Cameron, J., O’Connell, T., Evans, J., Marter, P., Taylor, G.M. (2017). Investigation of a Medieval Pilgrim Burial Excavated from the *Leprosarium* of St Mary Magdalen Winchester, UK. *PLoS Neglected Tropical Diseases* **11**: e0005186. DOI: 10.1371/journal.pntd.0005186.

Rolandsen, G.L., Arthur, P., Alexander, M. (2019). A tale of two villages: Isotopic insight into diet, economy, cultural diversity and agrarian communities in medieval (11^th^-15^th^ century CE) Apulia, Southern Italy. *Journal of Archaeological Science: Reports* **28**. DOI: 10.1016/j.jasrep.2019.102009.

Rose, H.A., Meadows, J., Bjerregaard, M. (2018). High-resolution dating of a Medieval multiple grave. *Radiocarbon* **60**: 1547-1559.

Rosvold, J., Halley, D.J., Hufthammer, A.K., Minagawa, M., Andersen, R. (2010). The rise and fall of wild boar in a northern environment: evidence from stable isotopes and subfossil finds. *Holocene* **20**:1113–1121.

Rumpelmayr, K. (2012). *Reconstructing diet by stable isotope analysis (d13C and d15N): Two case studies from Bronze Age and Early Medieval Lower Austria*. Unpublished PhD dissertation: Universität Wien.

Russell, N., Cook, G.T., Ascough, P., Barrett, J.H., Dugmore, A. (2011). Species specific marine radiocarbon reservoir effect: a comparison of DR values between Patella vulgata (limpet) shell carbonate and Gadus morhua (Atlantic cod) bone collagen. *Journal of Archaeological Science* **38**: 1008-1015.

Russell, N., Cook, G.T., Ascough, P., Dugmore, A. (2010). Spatial variation in the marine radiocarbon reservoir effect throughout the Scottish post-Roman to Late Medieval Period: North Sea values (500–1350 BP). *Radiocarbon* **52**: 1166-1181.

Ryan, S.A., Reynard, L.M., Crowley, Q.G., Snoeck, C. (2018). Early medieval reliance on the land and the local: An integrated multi-isotope study (87Sr/86Sr, δ18O, δ13C, δ15N) of diet and migration in Co. Meath, Ireland. *Journal of Archaeological Science* **98**: 59–71.

Sakai, Y. (2017). *Transition from the Late Roman Period to the Early Anglo-Saxon Period in the Upper Thames Valley Based on Stable Isotopes* Unpublished PhD dissertation: University of Oxford.

Salamon, M., Coppa, A., McCormick, M., Rubini, M., Vargiu, R., Tuross, N. (2008). The consilience of historical and isotopic approaches in reconstructing the medieval Mediterranean diet. *Journal of Archaeological Science* **35**: 1667-1672.

Salazar-García, D.C., Benítez De Lugo Enrich, L.B., Álvarez García, H.J., Benito Sánchez, M. (2013). Estudio diacrónico de la dieta de los pobladores antiguos de Terrinches (Ciudad Real) a partir del análisis de isótopos estables sobre restos óseos humanos. *Revista Española de Antropología Física* **34**: 6-14.

Salazar-García, D.C., Richards, M.P., Nehlich, O., Henry, A.G. (2014). Dental calculus is not equivalent to bone collagen for isotope analysis: a comparison between carbon and nitrogen stable isotope analysis of bulk dental calculus, bone and dentine collagen from same individuals from the Medieval site of El Raval (Alicante, Spain). *Journal of Archaeological Science* **47**: 70-77.

Salazar-García, D.C., Romero, A., García-Borja, P., Subirà, M.E., Richards, M.P. (2016). A combined dietary approach using isotope and dental buccal-microwear analysis of human remains from the Neolithic, Roman and Medieval periods from the archaeological site of Tossal de les Basses (Alicante, Spain). *Journal of Archaeological Science: Reports* **6**: 610-619.

Salesse, K., Dufour, É., Castex, D., Velemínský, P., Santos, F., Kuchařová, H., Jun, L., Brůžek, J. (2013). Life History of the Individuals Buried in the St. Benedict Cemetery (Prague, 15th–18th Centuries): Insights from 14C Dating and Stable Isotope (d13C, d15N, d18O) Analysis. *American Journal of Physical Anthropology* **151**: 202-214.

Salmi, A.K., Äikäs, T., Fjellström, M., Spangen, M. (2015). Animal offerings at the Sámi offering site of Unna Saiva – Changing religious practices and human–animal relationships. *Journal of Anthropological Archaeology* **40**: 10-22.

Salmi, A.K., Fjellström, M., Äikäs, T., Spangen, M., Núñez, M. (2020). Zooarchaeological and stable isotope evidence of Sámi reindeer offerings. *Journal of Archaeological Science: Reports* **29**: 102129. DOI: 10.1016/j.jasrep.2019.102129.

Sandias, M., Müldner, G. (2015). Diet and herding strategies in a changing environment: Stable isotope analysis of Bronze Age and Late Antique skeletal remains from Ya'amūn, Jordan. *Journal of Archaeological Science* **63**: 24-32.

Saragoça, P., Maurer, A.F. Šoberl, L., da Conceição Lopes, M., Alfenim, R., Leandro, I., Umbelino, C., Fernandes, T., Valente, M.J., Ribeiro, S., Santos, J.F., Janeiro, A.I., Dias Barrocas, C. (2016). Stable isotope and multi-analytical investigation of Monte da Cegonha: A Late Antiquity population in southern Portugal. *Journal of Archaeological Science: Reports* **9**: 728-742.

Sayle, K.L., Cook, G.T., Ascough, P.L., Gestsdóttir, H., Hamilton, W.D., McGovern, T.H. (2014). Utilization of δ13C, δ15N, and δ34S analyses to understand 14C dating anomalies within a Late Viking age community in Northeast Iceland. *Radiocarbon* **56**: 811-821.

Sayle, K.L., Cook, G.T., Ascough, P.L., Hastie, H.R., Einarsson, Á., McGovern, T.H., Hicks, M.T., Edwald, Á., Friðriksson, A. (2013). Application of 34S analysis for elucidating terrestrial, marine and freshwater ecosystems: Evidence of animal movement/husbandry practices in an early Viking community around Lake Mývatn, Iceland. *Geochimica et Cosmochimica Acta* **120**: 531-544.

Sayle, K.L., Hamilton, W.D., Cook, G.T., Ascough, P.L., Gestsdóttir, H., McGovern, T.H. (2016). Deciphering Diet and Monitoring Movement: Multiple Stable Isotope Analysis of the Viking Age Settlement at Hofstaðir, Lake Mývatn, Iceland. *American Journal of Physical Anthropology* **160**: 126-136.

Schäuble, A. (2006). *Ernährungsrekonstruktion dreier mittelalterlicher Bevölkerungen anhand der Analyse stabiler Isotope und Spurenelemente*. Unpublished PhD dissertation: Freie Universität Berlin.

Schuh, C., Makarewicz, C.A. (2016). Tracing residential mobility during the Merovingian period: An isotopic analysis of human remains from the Upper Rhine Valley, Germany. *American Journal of Physical Anthropology* **161**: 155-169.

Schutkowski, H., Herrmann, B., Bocherens, H., Grupe, G. (1999). Diet, Status and Decomposition at Weingarten: Trace Element and Isotope Analyses on Early Mediaeval Skeletal Material. *Journal of Archaeological Science* **26**: 675-685.

Schyman, J. (2012). *Proveniensbestämning av vikingatida hornmaterial - En studie utifrån stabila isotoper*. Unpublished MA dissertation: Stockholm University.

Scorrano, G., Brilli, M., Martínez-Labarga, C., Giustini, F., Pacciani, E., Chilleri, F., Scaldaferri, F., Gasbarrini, A., Gasbarrini, G., Rickards, O. (2014). Palaeodiet reconstruction in a woman with probable celiac disease: A stable isotope analysis of Bone remains from the Archaeological site of Cosa (Italy). *American Journal of Physical Anthropology* **154**: 349-356.

Sheridan, S.G., Gregoricka, L.A. (2015). Monks on the Move: Evaluating Pilgrimage to Byzantine St. Stephen’s Monastery Using Strontium Isotopes. *American Journal of Physical Anthropology* **158**: 581-591.

Sirignano, C., Sologestoa, I.G., Ricci, P., García-Collado, M.I., Altieri, S., Quirós Castillo, J.A., Lubritto, C. (2014). Animal husbandry during Early and High Middle Ages in the Basque Country (Spain). *Quaternary International* **346**: 138-148.

Skipityė, R., Lidén, K., Eriksson, G., Kozakaitė, J., Lauẑikas, R., Piličiauskienė, G., Jankauskas, R. (2020). Diet patterns in medieval to early modern (14^th^-20^th^ c.) coastal communities in Lithuania. *Anthropologischer Anzeiger*. DOI: 10.1127/anthranz/2020/1092

Smith, M.H., Smith, K.P., Frei, K.M. (2019). ‘Tangled up in Blue’: The Death, Dress and Identity of an Early Viking-Age Female Settler from Ketilsstaðir, Iceland. *Medieval Archaeology* **63**: 95-127.

Smrčka, V., Velemínský, P., Bůzek, F., Zocová, J. (2008). Stable C, N Isotopes in Human Skeletal Material from the Great Moravian Burial Site at Mikulčice-Kostelisko. In Velemínský, P., Poláček, L. (eds.), *Anthropological and epidemiological characterization of Great-Moravian population in connection with the social and economic structure*. Brno. 169-175.

Spangen, M., Fjellström, M. (2018). A fishy tale about a sheep and a dog – isotope studies and Medieval Sámi mobility and husbandry in inner Finnmark, Northern Norway. *Fennoscandia archaeologica* **35**: 3-17.

Speed, G., Walton Rogers, P., Budd, P., Clogg, P., Langston, J., Paterson, E. (2004). A Burial of a Viking Woman at Adwick-le-Street, South Yorkshire. *Medieval Archaeology* **48**: 51-90.

Spencer, R.K. (2008). *Testing hypotheses about di_use idiopathic skeletal hyperostosis (DISH) using stable isotope and aDNA analysis of late medieval British populations*. Unpublished PhD dissertation: Durham University.

Strott, N., Czermak, A., Grupe, G. (2008). Are biological correlates to social stratifi cation depicted in skeletal finds? Investigation of early medieval separate burial grounds in Bavaria. In Grupe, G., Peters, J. (eds.), *Skeletal Series and their Socio-economic Context*. Leidorf. 67-86.

Sveinbjörnsdóttir, Á., Heinemeier, J., Arneborg, J., Lynnerup, N., Ólafsson, G., Zoëga, G. (2010). Dietary reconstruction and reservoir correction of 14C dates on bones from pagan and early Christian graves in Iceland, *Radiocarbon* **52**: 682-696.

Symonds, L., Price, T.D., Keenleyside, A., Burton, J. (2014). Medieval Migrations: Isotope Analysis of Early Medieval Skeletons on the Isle of Man. *Medieval Archaeology* **58**: 1-20.

Tafuri, M.A., Goude, G., Manzi, G. (2018). Isotopic evidence of diet variation at the transition between classical and post-classical times in Central Italy. *Journal of Archaeological Science: Reports* **21**: 496-503.

Takken Beijersbergen, L.M., Fernandes, R., Mørkved, P.T., Hufthammer, A.K. (2021). Temporal and spatial variability of bone collagen stable carbon and nitrogen isotopic ratios of Norwegian reindeer. *Journal of Archaeological Science: Reports* **37**: 102890.

Taylor, G.M., Murphy, E.M, Mendum, T.A., Pike, A.W.G., Linscott, B., Wu, H., O’Grady, J., Richardson, H., O’Donovan, E., Troy, C., Stewart, G. (2018). Leprosy at the edge of Europe—Biomolecular, isotopic and osteoarchaeological findings from medieval Ireland. *PLoS One* **13**: e0209495. DOI: 10.1371/journal.pone.0209495.

Taylor, G.M., Tucker, K., Butler, R., Pike, A.W.G., Lewis, J., Roffey, S., Marter, P., Lee, O.Y., Wu, H.H.T., Minnikin, D.E., Besra, G.S., Singh, P., Cole, S.T., Stewart, G.R. (2013). Detection and Strain Typing of Ancient Mycobacterium leprae from a Medieval Leprosy Hospital. *PLoS One* **8**: e62406. DOI: 10.1371/journal.pone.0062406.

Tomczyk, J., Szostek, K., Lisowska-Gaczorek, A., Mnich, B., Zalewska, M., Trzeciecki, M., Olczak-Kowalczyk, D. (2020). Dental caries and isotope studies in the population of Radom (Poland) between the 11th and 19th centuries. *International Journal of Osteoarchaeology*. DOI: 10.1002/oa.2908

Tomczyk, J., Wierzbowski, H., Zalewska, M. (2015). Stable Isotope Record of Human and Sheep Enamel Carbonate from the Ancient Middle Euphrates Valley (Syria). *International Journal of Osteoarchaeology* **26**: 599-609.

Torino, M., Bodsen, J.L., Tarp, P., Rasmussen, K.L., Skytte, L., Nielsen, L., Schiavone, S., Terrasi, F., Passariello, I., Ricci, P., Lubritto, C. (2015). Convento di San Francesco a Folloni: the function of a Medieval Franciscan Friary seen through the burials. *Heritage Science* **27**. DOI: 10.1186/s40494-015-0056-z.

Toso, A., Alexander, M.M. (2018). Paleodietary reconstruction. *Setúbal Arqueológica* **17**: 203-206.

Toso, A., Gaspar, S., Bahna Da Silva, R., Garcia, S.J., Alexander, M.M. (2019). High status diet and health in Medieval Lisbon: a combined isotopic and osteological analysis of the Islamic population from São Jorge Castle, Portugal. *Archaeological and Anthropological Sciences* **11**: 3699–3716.

Trautmann, B., Wißing, C., Díaz-Zorita Bonilla, M., Bis-Worch, C., Bocherens, H. (2017). Reconstruction of Socioeconomic Status in the Medieval (14th–15th Century) Population of Grevenmacher (Luxembourg) Based on Growth, Development and Diet. *International Journal of Osteoarchaeology* **27**: 947-957.

Tütken, T., Langenegger, E., Wild, W. (2008). Einheimisch oder fremd? Isotopenanalyse eines Frauenskelettes des 9. Jahrhunderts n. Chr. Aus Elsau, Kanton Zürich, Schweiz. *Anthropologischer Anzeiger* **66**: 19-50.

Van der Jagt, I.M.M., Kootker, L.M., van Kolfschoten, T., Kars, H., Davies, G.R. (2012). An insight into animal exchange in Early Medieval Oegstgeest: a combined archaeozoological and isotopic approach. In Raemaekers, D.C.M., Esser, E., Lauwerier, R.C.G.M., Zeiler, J.T. (eds.), *A Bouquet of Archaeological Studies. Essays in Honour of Wietske Prummel*. Groningen. 139-149.

Van der Sluis, L.G., Hollund, H.I., Kars, H., Sandvik, P.U., Denham, S.D. (2016). A palaeodietary investigation of a multi-period churchyard in Stavanger, Norway, using stable isotope analysis (C, N, H, S) on bone collagen. *Journal of Archaeological Science: Reports* **9**: 120-133.

Van der Sluis, L.G., Reimer, P.J., Lynnerup, N. (2015). Investigating intra-individual dietary changes and 14C ages using high-resolution δ13C and δ15N isotope ratios and 14C ages obtained from dentine increments. *Radiocarbon* **57**: 665-677.

Van Strydonck, M., Ervynck, A., Vandenbruaene, M., Boudin, M. (2009). Anthropology and 14C Analysis of Skeletal Remains from Relic Shrines: An Unexpected Source of Information for Medieval Archaeology. *Radiocarbon* **51**: 569-577.

Varano, S., De Angelis, F., Battistini, A., Brancazi, L., Pantano, W., Ricci, P., Romboni, M., Catalano, P., Gazzaniga, V., Lubritto, C., Santangeli Valenzani, R., Martínez-Labarga, C., Rickards, O. (2020). The edge of the Empire: diet characterization of medieval Rome through stable isotope analysis. *Archaeological and Anthropological Science* **12**. DOI: 10.1007/s12520-020-01158-3

Vidal-Ronchas, R., Šikanjić, P.R., Premužić, Z., Lightfoot, E. (2019). Diet, sex, and social status in the Late Avar period: stable isotope investigations at Nuštar cemetery, Croatia. *Archaeological and Anthropological Science* **11**: 1727-1737.

Viva, S., Fabbri, P.F., Ricci, P., Bianchi, G., Hodges, R., Lubritto, C. (2021). Project nEU-Med. The Contribution of Isotopic Analysis in the Differential Diagnosis of Anemia, the Case of the Medieval Cemetery of Vetricella (Scarlino, GR) in Tuscany. *Environmental Archaeology*. DOI: 10.1080/14614103.2020.1867290

Voas, M.R., Killgrove, K., Bethad, J.D., Tykot, R.H., Nyaradi, Z., Gonciar, A. (2018). Milk and Honey: Isotopic Reconstruction of Infant Weaning in Medieval Transylvania. *American Journal of Physical Anthropology* **165**. Poster.

Vohberger, M.A. (2011). *Lokal oder Eingewandert? Interpretationsmöglichkeiten und Grenzen lokaler Strontium- und Sauerstoffisotopensignaturen am Beispiel einer Altgrabung in Wenigumstadt*. Unpublished PhD dissertation: Ludwig-Maximilians.Universität München.

Von Heyking, K., Zintl, S. (2015). The Early-Merovingian Cemetery in München-Perlach (Bavaria). Analysing Skeletal Morphology, Health and Disease and Strontium Isotope Ratios. *Interdisciplinaria Archaeologica* **6**. https://www.iansa.eu/papers/IANSA-2016-01-heyking_onlinefirst.pdf

Vytlačil, Z., Kaupová, S., Lefebvre, A., Velemínský, P., Brůžek, J. (2018). A time of change: dietary reconstruction of the Merovingian cemetery of Norroy-le-Veneur, France. *Anthropologischer Anzeiger* **75**: 325-338.

Vytlačil, Z., Kaupová, S., Jílková, M., Poláček, L., Ackerman, L., Velemínský, P. (2021). Residential mobility in Great Moravia: strontium isotope analysis of a population sample from the early medieval site of Mikulčice-Valy (ninth–tenth centuries). *Archaeological and Anthropological Sciences* **13**. DOI: 10.1007/s12520-020-01247-3

Wahl, J., Cipollini, G., Coia, V., Francken, M., Harvati-Papatheodorou, K., Kim, M., Maixner, F., O’Sullivan, N., Price, T.D., Quast, D., Speith, N., Zink, A. (2014). Neue Erkenntnisse zur frühmittelalterlichen Separatgrablege von Niederstotzingen, Kreis Heidenheim. *Fundberichte aus Baden-Württemberg* **34**: 341-390.

Walser III, J.W., Kristjánsdóttir, S., Gröcke, D.R., Gowland, R.L., Jakob, T., Nowell, G.M., Ottley, C.J., Montgomery, J. (2020). At the world's edge: Reconstructing diet and geographic origins in medieval Iceland using isotope and trace element analyses. *American Journal of Physical Anthropology* **171**: 142-163.

Walter, B.S., DeWitte, S.N., Dupras, T., Beaumont, J. (2020). Assessment of nutritional stress in famine burials using stable isotope analysis. American Journal of Physical Anthropology 172: 214-226.

Whitmore, K.M., Dupras, T.L., Williams, L.J., Skipitytė, R., Schultz, J.J., Jankauskas, R. (2019). Stable carbon and nitrogen isotope inter- and intra-individual dietary reconstruction from the late 14th to early 18th century site of Alytus, Lithuania. *American Journal of Physical Anthropology* **168**: 279-291.

Wiedermann, F.B., Bocherens, H. (1997). Spurenelement- und Isotopenanalyse in archäologischem Knochen im Vergleich (am Beispielder mittelalterlichen Skelettserie von Weingarten, Deutschland). *Anthropologischer Anzeiger* **55**: 147-154.

Wilhelmson, H., Price, T.D. (2017). Migration and integration on the Baltic island of Öland in the Iron Age. *Journal of Archaeological Science: Reports* **12**: 183-196.

Winter-Schuh, C., Makarewicz, C.A. (2019). Isotopic evidence for changing human mobility patterns after the disintegration of the Western Roman Empire at the Upper Rhine. *Archaeological and Anthropological Sciences* **11**: 2937-2955.

Wong, M., Brandt, J.R., Ahrens, S., Jaouen, K., Bjørnstad, G. Naumann, E, Wenn, C.C., Kiesewetter, H., Laforest, C., Hagelberg, E., Lam, V.C., Richards, M.P. (2018). Pursuing pilgrims: Isotopic investigations of Roman and Byzantine mobility at Hierapolis, Turkey. *Journal of Archaeological Science: Reports* **17**: 520-528.

Yoder, C. (2010). Diet in medieval Denmark: a regional and temporal comparison. *Journal of Archaeological Science* **37**: 2224-2236.

Yoder, C. (2012). Let them eat cake? Status-based differences in diet in medieval Denmark. *Journal of Archaeological Science* **39**: 1183-1193.

Zechini, M., Killgrove, K., Melisch, C., Powers, N., Jungklaus, B. (2018). Diet and Disease in Medieval Berlin: Dental Pathology Data from the Medieval Cemetery of Petriplatz. *American Journal of Physical Anthropology* **165**. Poster.
