# Supplementary material for: Presenting the Compendium Isotoporum Medii Aevi, a Multi-Isotope Database for Medieval Europe
Source: Sci Data. 2022 Jun 21;9:354. doi: 10.1038/s41597-022-01462-8 (PMC9213510; doi:10.1038/s41597-022-01462-8)
Supplement: Supplementary file 4 — Supplementary Information File 4 [file 41597_2022_1462_MOESM4_ESM.docx]

**Supplementary Information File S4: Bayesian modeling**

**Pandora & IsoMemo modeling**

The illustrative case studies described in the main text employed R-based[^1^](https://www.zotero.org/google-docs/?cpB2Wg) modeling tools (TimeR, AverageR, OperatoR, KernelTimeR and LocateR) developed within the Pandora & IsoMemo initiatives and made available via Shiny[^2^](https://www.zotero.org/google-docs/?cNIHRn) graphical interfaces (https://isomemoapp.com/). Below a description of these models is given and the full model settings are given in Supplementary Information File S5. The R code for these apps can be downloaded from <https://isomemoapp.com> and apps run locally using RStudio[^3^](https://www.zotero.org/google-docs/?GTTKbm).

*TimeR*

‘TimeR’ is a model available within the IsoMemo app listed at <https://isomemoapp.com/> within the menu options “Modeling”.

‘TimeR’ is a Bayesian geostatistical model that estimates the expected value of a “dependent” variable across time and space. The underlying model formula is:

Y_i = g(long_i, lat_i, time_i) + gamma_site_i + epsilon_i

gamma_site_i is a random effect with ~ N(0, tau^2)

where g()- is a three dimensional (spatial coordinates plus time) smooth function using a so-called thin plate regression spline (“tprs”[^4^](https://www.zotero.org/google-docs/?BluQz8)) and epsilon follows a normal distribution N(0, sigma^2). As the time variable is measured with uncertainty, a Metropolis-Hastings step is employed to account for it (further details given in Groß[^5^](https://www.zotero.org/google-docs/?Gz5s0J)). The model was employed to model spatiotemporal variations in human bone collagen isotopic values for early medieval Europe and for the site of Rome (Fig. 4-6). Human data was filtered to include only non-elite adult individuals with bone and tooth collagen C:N atomic ratios within the acceptance interval given by DeNiro[^6^](https://www.zotero.org/google-docs/?zhKYLr).

*AverageR*

‘AverageR’ is a model available within the IsoMemo app listed at <https://isomemoapp.com/> within the menu options “Modeling”.

‘AverageR’ is a Bayesian geostatistical model that estimates the expected value of the “dependent” variable across space. ‘AverageR’ is similar to ‘TimeR’ but does not include time dependence[^5,7^](https://www.zotero.org/google-docs/?cliWLv). The model was employed to map the spatial distribution of δ^13^C and δ^15^N mean and prediction error values (double the square root of the following sum: the square of the predicted standard error of the mean plus the square of the predicted population standard deviation) for bone collagen from medieval domesticated animals (Fig. 3) and to establish reference baselines for mobility studies (see below, LocateR). For the animals, we modeled separately isotopic values for cattle and sheep/goat *versus* pig and chicken with acceptable C:N atomic ratios[^6^](https://www.zotero.org/google-docs/?hsE1EA).

*OperatoR*

‘OperatoR’ is available within the IsoMemo app listed at <https://isomemoapp.com/> within the menu options “Modeling”.

OperatoR was used to plot the differences among maps generated using AverageR. This difference was calculated by subtracting two-dimensional posterior estimates of the time sliced g()-smooth, i.e. the expected values for all considered locations for a fixed date. OperatoR was used to show the temporal differences in early medieval diets for two different time-slices (Fig. 4). ‘TimeR’ was used to generate spatial models in three different time-slices (200-500-800 CE) followed by the use of OperatoR to map differences.

*KernelTimeR*

‘KernelTimeR’ is available within the IsoMemo app listed at <https://isomemoapp.com/> within the menu options “Modeling”.

‘KernelTimeR’ is a 3-dimensional spatiotemporal kernel density estimator[^8^](https://www.zotero.org/google-docs/?AkRnxA). It was employed to show research gaps in early medieval isotopic studies and to assess temporal variability in human mobility patterns (Fig. 8). Mobile *versus* non-mobile individuals were identified by comparing individual values with a ^87^Sr/^86^Sr and δ^18^O_phosphate_ tooth enamel baseline for Britain[^9,10^](https://www.zotero.org/google-docs/?IaOt55) modeled using ‘AverageR’. Tooth enamel carbonate values reported relative to VPDB standard were converted into values relative to the VSMOW standard (δ^18^O_VSMOW_ = δ^18^O_VPDB_ * 1.03092 + 30.92). These were then converted into phosphate values using the expression (δ^18^O_phosphate_ = δ^18^O_carbonate_ *1.0322 - 9.6849) reported by Chenery and colleagues[^11^](https://www.zotero.org/google-docs/?vtowhX). Individuals were classified as mobile if their ^87^Sr/^86^Sr or δ^18^O tooth values were outside baseline ranges. For ^87^Sr/^86^Sr we considered the modeled 2-sigma range while for δ^18^O we used a conservative uncertainty of 1‰ to account for previously reported variability arising from different factors (e.g. diagenesis, cooking, etc.)[^12,13^](https://www.zotero.org/google-docs/?b0RKkM).

*LocateR*

‘LocateR’ is available within the IsoMemo app listed at <https://isomemoapp.com/> within the menu options “Modeling”.

Given a 2-dimensional grid of locations and corresponding estimates of mean and residual error from the ‘TimeR’ or ‘AverageR’ models (here the baseline spatial variations in oxygen and strontium isotopes), for a new given value of the dependent variable (here the oxygen and strontium isotope values for an individual), ‘LocateR’ assigns a density value to each location[^14^](https://www.zotero.org/google-docs/?wNYkN6). The model was employed to show probable dwelling locations (and likely places of origin for early formed teeth) for four different medieval individuals buried in England. Human enamel stable oxygen values were converted into phosphate values relative to the VSMOW standard following the same procedure as described in the previous paragraph. Conversion of these into δ^18^O for ingested water (δ^18^O_dw_ = δ^18^O_phosphate_ * 1.55 – 33.49) followed Pollard *et al.*’s formula[^15^](https://www.zotero.org/google-docs/?rXPfcg) .We used ‘AverageR’ to establish a baseline for water δ^18^O using the Cluster-based Water Isotope Prediction Model (RCWIP), which relies on modern measurements from the Global Network of Isotopes in Precipitation (GNIP)[^16^](https://www.zotero.org/google-docs/?sjWckW) and a ^87^Sr/^86^Sr and δ^18^O_phosphate_ tooth enamel baseline for Britain[^9,10^](https://www.zotero.org/google-docs/?VUgwir).

**ReSources**

‘ReSources’ is an app listed at <https://isomemoapp.com/>.

‘ReSources’ is a software used to define Bayesian mixing models that can be employed for isotope-based diet reconstruction. This software is an upgraded version of the Bayesian software FRUITS[^17^](https://www.zotero.org/google-docs/?iD2vzk). We employed the model to quantify caloric and macronutrient contribution of each food source within human diets in the area of Rome across three time-slices: 200 CE, 500 CE, and 800 CE. The model employs a random effects structure on the categorical covariate "Time" with levels 200 CE, 500 CE, and 800 CE. In contrast to a model without covariates, the Dirichlet prior values of the source contribution parameters ("alpha") are not fixed but come from a distribution with common mean and standard deviation for each factor level (200 CE, 500 CE, 800 CE) and source group.

Although the model has been employed in recent years to reconstruct past human diets[^18–20^](https://www.zotero.org/google-docs/?B4aDei), the chosen food categories were often very broad (e.g. following chemical and/or habitat classifications such as C_3_/C_4_ plants, C_3_ animals, marine animals, etc.). In our case-study, we relied on archaeobotanical, zooarchaeological and historical evidence to define the following main food groups for Roman and medieval populations at the city of Rome: Wheat; Barley; C_4_ Cereals (i.e. millet or sorghum); Pulses; Other C_3_ Plants (i.e. Vegetables, fruit, nuts); Cattle; Sheep/Goat; Pig; Poultry; Marine Sources; Freshwater Sources. Missing from the above, given a lack of isotopic references, is olive oil whose dietary contributions will likely fall within modeling results among C_3_ plants.

Human mean isotopic values for Rome (Lat. 41.9; Long. 12.5) were modeled using TimeR from bone collagen measurements available in the CIMA and IsoArcH[^21^](https://www.zotero.org/google-docs/?BMcVJG) databases. We considered three separate time slices (200 CE, 500 CE, and 800 CE) with the following results: 200 CE: δ^13^C=-19.14±0.11‰, δ^15^N=10.08±0.11‰; 500 CE: δ^13^C=-19.24±0.2‰, δ^15^N=9.57±0.23‰; 800 CE: δ^13^C=-19.05±0.38‰, δ^15^N=8.6±0.23‰. As for the isotopic food baseline (tab. S4.1 below), wheat, barley and pulses isotopic values were obtained from the O’Connell *et al.* publication on Portus[^22^](https://www.zotero.org/google-docs/?SsjCXU). Given the lack of direct isotopic measurements on archaeological fruit and vegetables, isotopic values for the ‘other C_3_ plants’ were calculated by applying a trophic isotopic offset correction to herbivore mean bone collagen carbon and nitrogen stable isotope values (δ^13^C=-4‰; δ^15^N=-3.5‰,[^23,24^](https://www.zotero.org/google-docs/?muAu7l)) between these and C_3_ plants. Lacking coeval isotopic values on C_4_ cereals, we employed isotopic measurements from two sites in Bronze Age Greece[^25^](https://www.zotero.org/google-docs/?AwHzdM), as reported in the IsoArcH database. As for food products from cattle, sheep/goat and pig the isotopic references given below were modelled using ‘TimeR’ for Rome (Lat. 41.9; Long. 12.5). For poultry, given a limited amount of data, we calculated the mean and standard deviation for isotopic values available from CIMA[^19,22,26–33^](https://www.zotero.org/google-docs/?ZppiTE). A similar approach was employed for Mediterranean marine sources by combining CIMA and IsoArcH data[^22,30,34–36^](https://www.zotero.org/google-docs/?DDcuXO). For freshwater sources, we collected medieval measurements from Italy[^32^](https://www.zotero.org/google-docs/?LHSezH), Switzerland[^37^](https://www.zotero.org/google-docs/?YAm6Wd) and France[^38^](https://www.zotero.org/google-docs/?ZTiOxV). Food sources isotopic values were the following:

|  | 200 CE δ^13^C | 500 CE δ^13^C | 800 CE δ^13^C |
| --- | --- | --- | --- |
| Charred Wheat remains | -23.18±0.87‰ | -22.07±0.96‰ | -22.45±0.35‰ |
| Charred Barley remains | -24.4±0.7‰ | -22±0.4‰ | -23.46±0.15‰ |
| Charred C_4_ Cereals remains | -10.4±0.27‰ | -10.4±0.27‰ | -10.4±0.27‰ |
| Charred Pulses remains | -24±1.3‰ | -26.5±0.4‰ | -21.87±0.75‰ |
| Other C_3_ as trophic isotopic offset towards herbivore bone collagen | -24.3±0.4‰ | -24±0.3‰ | -24±0.3‰ |
| Cattle bone collagen | -20.2±0.34‰ | -19.76±0.15‰ | -19.75±0.29‰ |
| Sheep/Goat bone collagen | -20.35±0.19‰ | -20.31±0.23‰ | -20.25±0.17‰ |
| Pig bone collagen | -20.15±0.12‰ | -19.88±0.07‰ | -20.16±0.11‰ |
| Poultry bone collagen | -18.7±2‰ | -18.7±2‰ | -18.7±2‰ |
| Marine Sources bone collagen | -11.3±3‰ | -11.3±3‰ | -11.3±3‰ |
| Freshwater Sources bone collagen | -23.39±1.3‰ | -23.39±1.3‰ | -23.39±1.3‰ |

|  | 200 CE δ^15^N | 500 CE δ^15^N | 800 CE δ^15^N |
| --- | --- | --- | --- |
| Charred Wheat remains | 8.53±3.11‰ | 6.76±3.84‰ | 4.3±0.28‰ |
| Charred Barley remains | 6.1±1.3‰ | 6.9±0.4‰ | 5.63±0.97‰ |
| Charred C_4_ Cereals remains | 6.8±2.74‰ | 6.8±2.74‰ | 6.8±2.74‰ |
| Charred Pulses remains | 2.5±0.6‰ | 2.5±0.6‰ | 2.4±0.46‰ |
| Other C_3_ as trophic isotopic offset towards herbivore bone collagen | 2.1±0.6‰ | 2±0.8‰ | 2.3±0.6‰ |
| Cattle bone collagen | 5.96±0.41‰ | 5.99±0.77‰ | 6.27±0.43‰ |
| Sheep/Goat bone collagen | 5.29±0.41‰ | 4.94±0.25‰ | 5.32±0.39‰ |
| Pig bone collagen | 6.34±0.24‰ | 6.13±0.16‰ | 5.98±0.34‰ |
| Poultry bone collagen | 7.6±2.1‰ | 7.6±2.1‰ | 7.6±2.1‰ |
| Marine Sources bone collagen | 10.7±1.9‰ | 10.7±1.9‰ | 10.7±1.9‰ |
| Freshwater Sources bone collagen | 7.98±1.88‰ | 7.98±1.88‰ | 7.98±1.88‰ |

Tab. S4.1. Isotopic values for food remains and time-slice employed in Bayesian dietary modeling. These do not include yet corrections for offsets between edible tissues and food remains (e.g. muscle meat protein or lipids vs. bone collagen collagen). Corrected values are given in Table Tab. S4.2.

To obtain δ^13^C and δ^15^N values for food macronutrient components (carbs/lipids vs. protein) we applied offset corrections between the measured material (e.g. bone collagen) and the edible nutritional component of the respective food group (muscle meat protein) (Tab. S4.2, below). We applied the following offset corrections with uncertainties for macronutrient isotopic values rounded up to multiples of 0.5[^18^](https://www.zotero.org/google-docs/?H7CoDG) (updated in Soncin *et al.*[^39^](https://www.zotero.org/google-docs/?ID5Ia3)): Plants: Δ^13^C_protein-bulk_=-2‰, Δ^13^C_carbohydrates-bulk_=+0.5‰, Δ^15^N_protein-bulk_=0‰; terrestrial animals: Δ^13^C_protein-collagen_= -2‰, Δ^13^C_lipids-collagen_= -8‰, Δ^15^N_protein-collagen_=0‰; aquatic animals: Δ^13^C_protein-collagen_=-1‰, Δ^13^C_lipids-collagen_=-7‰, Δ^15^N_protein-collagen_=+1.5‰). Below a list of isotopic reference values employed in modeling:

|  | 200 CE δ^13^C Protein | 200 CE δ^13^C Lipids/Carbohydrates | 200 CE δ^15^N Protein |
| --- | --- | --- | --- |
| Wheat | -25.2±2‰ | -22.7±2‰ | 8.5±4.5‰ |
| Barley | -26.4±2‰ | -23.9±2‰ | 6.1±2.5‰ |
| C_4_ Cereals | -12.4±1.5‰ | -9.9±1.5‰ | 6.8±4.0‰ |
| Pulses | -26.0±2.5‰ | -23.5±2.5‰ | 2.5±2‰ |
| Other C_3_ Plants | -26.3±1.5‰ | -23.8±1.5‰ | 2.1±2‰ |
| Cattle | -22.2±1.5‰ | -28.2±1.5‰ | 6±1.5‰ |
| Sheep/Goat | -22.4±1.5‰ | -28.4±1.5‰ | 5.3±1.5‰ |
| Pig | -22.2±1.5‰ | -28.2±1.5‰ | 6.3±1.5‰ |
| Poultry | -20.7±3‰ | -26.7±3‰ | 7.6±3.5‰ |
| Marine Sources | -12.3±4‰ | -18.3±4‰ | 12.2±3‰ |
| Freshwater Sources | -24.4±2.5‰ | -30.4±2.5‰ | 9.5±3‰ |

|  | 500 CE δ^13^C Protein | 500 CE δ^13^C Lipids/Carbohydrates | 500 CE δ^15^N Protein |
| --- | --- | --- | --- |
| Wheat | -24.1±2‰ | -21.6±2‰ | 6.8±5‰ |
| Barley | -24.0±1‰ | -21.5±1‰ | 6.9±1‰ |
| C_4_ Cereals | -12.4±1.5‰ | -9.9±1.5‰ | 6.8±4.0‰ |
| Pulses | -28.5±1‰ | -26±1‰ | 2.5±2‰ |
| Other C_3_ Plants | -26.0±1.5‰ | -23.5±1.5‰ | 2±2‰ |
| Cattle | -21.8±1.5‰ | -27.8±1.5‰ | 6.0±2‰ |
| Sheep/Goat | -22.3±1.5‰ | -28.3±1.5‰ | 4.9±1.5‰ |
| Pig | -21.9±1.5‰ | -27.9±1.5‰ | 6.1±1.5‰ |
| Poultry | -20.7±3‰ | -26.7±3‰ | 7.6±3.5‰ |
| Marine Sources | -12.3±4‰ | -18.3±4‰ | 12.2±3‰ |
| Freshwater Sources | -24.4±2.5‰ | -30.4±2.5‰ | 9.5±3‰ |

|  | 800 CE δ^13^C Protein | 800 CE δ^13^C Lipids/Carbohydrates | 800 CE δ^15^N Protein |
| --- | --- | --- | --- |
| Wheat | -24.5±1.5‰ | -22.0±1.5‰ | 4.3±1.5‰ |
| Barley | -25.5±1.5‰ | -23.0±1.5‰ | 5.6±2‰ |
| C_4_ Cereals | -12.4±1.5‰ | -9.9±1.5‰ | 6.8±4.0‰ |
| Pulses | -23.9±2‰ | -21.4±2‰ | 2.4±1.5‰ |
| Other C_3_ Plants | -26±1.5‰ | -23.5.0±1.5‰ | 2.3±2‰ |
| Cattle | -21.8±1.5‰ | -27.8±1.5‰ | 6.3±1.5‰ |
| Sheep/Goat | -22.3±1.5‰ | -27.8±1.5‰ | 5.3±1.5‰ |
| Pig | -22.2±1.5‰ | -28.2±1.5‰ | 6±1.5‰ |
| Poultry | -20.7±3‰ | -26.7±3‰ | 7.6±3.5‰ |
| Marine Sources | -12.3±4‰ | -18.3±4‰ | 12.2±3‰ |
| Freshwater Sources | -24.4±2.5‰ | -30.4±2.5‰ | 9.5±3‰ |

Tab.S4.2. Corrected isotopic food macronutrients values as employed in dietary modeling.

Within the Bayesian mixing model we employed macronutrient caloric concentration values as reported in Fernandes *et al*.[^17^](https://www.zotero.org/google-docs/?d1XvCd) but with doubled uncertainty values. These were for plant foods: protein: 10±5%; carbs/lipids 90±5%%, terrestrial animals: protein: 30±5%; carbs/lipids 70±5%, and aquatic animals: protein: 65±10%; carbs/lipids 35±10%.

The Bayesian mixing model also accounted for isotopic offsets between diet and human tissues and dietary routing mechanisms. Bone collagen δ^15^N was assumed to fully derive from dietary protein with an isotopic offset of 5.5±0.5‰[^18^](https://www.zotero.org/google-docs/?zbohgD). For bone collagen δ^13^C we considered an offset of 4.8±0.5‰ towards food values and that the isotopic signal was routed: 74±4% from dietary protein and 26±4% from carbohydrates/lipids[^40^](https://www.zotero.org/google-docs/?yXPBqS).

To improve the precision of dietary estimates we employed prior dietary information from zooarchaeological, archaeobotanical and historical evidence relative to ancient Rome (Fig. 6). We considered that, in farming societies plants were the main caloric sources for lower status individuals. We set the following constraint in the Bayesian model: Wheat+barley+C_4_ cereals+pulses>57% of the caloric contribution. This value is the mean caloric contribution from starches consumed in Mediterranean countries between 1960–1965 (table 14.2 reported in Garnsey and Scheidel[^41^](https://www.zotero.org/google-docs/?9EZENu)).

We collected archaeobotanical evidence from central and southern Italy[^27,42–46^](https://www.zotero.org/google-docs/?7isWTr), and from this ordered plant starch dietary contributions as follows: Wheat>Pulses>Barley>Millet. This data plus that from written sources[^47^](https://www.zotero.org/google-docs/?5EQVdO) shows a temporal decrease in wheat consumption. The consumption of C_4_ cereals increased during the medieval period for rural Italian populations[^47^](https://www.zotero.org/google-docs/?RX7LN8). During the Late Roman and early medieval period the consumption of animal food sources decreased and populations relied increasingly on cereals, legumes, and other plants[^47^](https://www.zotero.org/google-docs/?7lO2jZ).

The zooarchaeological evidence[^48–50^](https://www.zotero.org/google-docs/?APA8r2) shows that pig remains were the most abundant for all periods although this decreased through time in favor of sheep/goat and to a lesser extent cattle.

Dietary modeling results are shown in Fig. 6 and summarized in Supplementary Information file S6. The latter also includes dietary estimates for modern Italians. For this we employed data from FAO food balance sheets (<http://www.fao.org/faostat/en/#data/FBS>) for Italy during 2018 and from 1994–96[^51^](https://www.zotero.org/google-docs/?gqftE1). These results are given in Supplementary Information File S6. These were compared with Bayesian dietary estimates for Roman and medieval populations at Rome but only for sources available in the past (normalized values).

The full model is available within the MATILDA repository (<https://pandoradata.earth/dataset/cima-compendium-isotoporum-medii-aevi/resource/6dd3aeab-01d6-4f0e-8e82-bbcef5ac507c>) as a R file (.RData). All model inputs are also given in Supplementary Information File 5.

**References**

[1. R Core Team. *R: A Language and Environment for Statistical Computing*. (R Foundation for Statistical Computing, 2021).](https://www.zotero.org/google-docs/?cfMyDP)

[2. Chang, W., Cheng, J., Allaire, J., Xie, Y. & McPherson, J. Shiny: web application framework for R. *R Package Version* **1**, 2017 (2017).](https://www.zotero.org/google-docs/?cfMyDP)

[3. RStudio Team. *RStudio: Integrated Development Environment for R*. (RStudio, PBC., 2020).](https://www.zotero.org/google-docs/?cfMyDP)

[4. Wood, S. N. Thin plate regression splines. *J. R. Stat. Soc. Ser. B Stat. Methodol.* **65**, 95–114 (2003).](https://www.zotero.org/google-docs/?cfMyDP)

[5. Groß, M. Modeling body height in prehistory using a spatio-temporal Bayesian errors-in variables model. *AStA Adv. Stat. Anal.* **100**, 289–311 (2016).](https://www.zotero.org/google-docs/?cfMyDP)

[6. DeNiro, M. J. Postmortem preservation and alteration of in vivo bone collagen isotope ratios in relation to palaeodietary reconstruction. *Nature* **317**, 806–809 (1985).](https://www.zotero.org/google-docs/?cfMyDP)

[7. Cubas, M. *et al.* Latitudinal gradient in dairy production with the introduction of farming in Atlantic Europe. *Nat. Commun.* **11**, 2036 (2020).](https://www.zotero.org/google-docs/?cfMyDP)

[8. Wand, M. P. & Jones, M. C. Multivariate plug-in bandwidth selection. *Comput. Stat.* **9**, 97–116 (1994).](https://www.zotero.org/google-docs/?cfMyDP)

[9. Evans, J. A., Montgomery, J., Wildman, G. & Boulton, N. Spatial variations in biosphere 87Sr/86Sr in Britain. *J. Geol. Soc.* **167**, 1–4 (2010).](https://www.zotero.org/google-docs/?cfMyDP)

[10. Pellegrini, M., Pouncett, J., Jay, M., Pearson, M. P. & Richards, M. P. Tooth enamel oxygen “isoscapes” show a high degree of human mobility in prehistoric Britain. *Sci. Rep.* **6**, 34986 (2016).](https://www.zotero.org/google-docs/?cfMyDP)

[11. Chenery, C. A., Pashley, V., Lamb, A. L., Sloane, H. J. & Evans, J. A. The oxygen isotope relationship between the phosphate and structural carbonate fractions of human bioapatite. *Rapid Commun. Mass Spectrom.* **26**, 309–319 (2012).](https://www.zotero.org/google-docs/?cfMyDP)

[12. Lightfoot, E. & O’Connell, T. C. On the Use of Biomineral Oxygen Isotope Data to Identify Human Migrants in the Archaeological Record: Intra-Sample Variation, Statistical Methods and Geographical Considerations. *PLOS ONE* **11**, e0153850 (2016).](https://www.zotero.org/google-docs/?cfMyDP)

[13. Tuross, N., Reynard, L. M., Harvey, E., Coppa, A. & McCormick, M. Human skeletal development and feeding behavior: the impact on oxygen isotopes. *Archaeol. Anthropol. Sci.* **9**, 1453–1459 (2017).](https://www.zotero.org/google-docs/?cfMyDP)

[14. Wang, X. *et al.* The Circulation of Ancient Animal Resources Across the Yellow River Basin: A Preliminary Bayesian Re-evaluation of Sr Isotope Data From the Early Neolithic to the Western Zhou Dynasty. *Front. Ecol. Evol.* **9**, 16 (2021).](https://www.zotero.org/google-docs/?cfMyDP)

[15. Pollard, A. m., Pellegrini, M. & Lee-Thorp, J. a. Technical note: Some observations on the conversion of dental enamel δ18Op values to δ18Ow to determine human mobility. *Am. J. Phys. Anthropol.* **145**, 499–504 (2011).](https://www.zotero.org/google-docs/?cfMyDP)

[16. Terzer, S., Wassenaar, L. I., Araguás-Araguás, L. J. & Aggarwal, P. K. Global isoscapes for δ18O and δ2H in precipitation: improved prediction using regionalized climatic regression models. *Hydrol. Earth Syst. Sci.* **17**, 4713–4728 (2013).](https://www.zotero.org/google-docs/?cfMyDP)

[17. Fernandes, R., Millard, A. R., Brabec, M., Nadeau, M.-J. & Grootes, P. Food Reconstruction Using Isotopic Transferred Signals (FRUITS): A Bayesian Model for Diet Reconstruction. *PLoS ONE* **9**, e87436 (2014).](https://www.zotero.org/google-docs/?cfMyDP)

[18. Fernandes, R., Grootes, P., Nadeau, M.-J. & Nehlich, O. Quantitative diet reconstruction of a Neolithic population using a Bayesian mixing model (FRUITS): The case study of Ostorf (Germany). *Am. J. Phys. Anthropol.* **158**, 325–340 (2015).](https://www.zotero.org/google-docs/?cfMyDP)

[19. Varano, S. *et al.* The edge of the Empire: diet characterization of medieval Rome through stable isotope analysis. *Archaeol. Anthropol. Sci.* **12**, 196 (2020).](https://www.zotero.org/google-docs/?cfMyDP)

[20. Bownes, J. M., Ascough, P. L., Cook, G. T., Murray, I. & Bonsall, C. Using Stable Isotopes and a Bayesian Mixing Model (FRUITS) to Investigate Diet at the Early Neolithic Site of Carding Mill Bay, Scotland. *Radiocarbon* **59**, 1275–1294 (2017).](https://www.zotero.org/google-docs/?cfMyDP)

[21. Salesse, K. *et al.* IsoArcH.eu: An open-access and collaborative isotope database for bioarchaeological samples from the Graeco-Roman world and its margins. *J. Archaeol. Sci. Rep.* **19**, 1050–1055 (2018).](https://www.zotero.org/google-docs/?cfMyDP)

[22. O’Connell, T. C. *et al.* Living and dying at the Portus Romae. *Antiquity* **93**, 719–734 (2019).](https://www.zotero.org/google-docs/?cfMyDP)

[23. Lee-Thorp, J. A., Sealy, J. C. & van der Merwe, N. J. Stable carbon isotope ratio differences between bone collagen and bone apatite, and their relationship to diet. *J. Archaeol. Sci.* **16**, 585–599 (1989).](https://www.zotero.org/google-docs/?cfMyDP)

[24. Hedges, R. E. M. & Reynard, L. M. Nitrogen isotopes and the trophic level of humans in archaeology. *J. Archaeol. Sci.* **34**, 1240–1251 (2007).](https://www.zotero.org/google-docs/?cfMyDP)

[25. Nitsch, E. *et al.* A bottom-up view of food surplus: using stable carbon and nitrogen isotope analysis to investigate agricultural strategies and diet at Bronze Age Archontiko and Thessaloniki Toumba, northern Greece. *World Archaeol.* **49**, 105–137 (2017).](https://www.zotero.org/google-docs/?cfMyDP)

[26. Baldoni, M. *et al.* Archaeo-biological reconstruction of the Italian medieval population of Colonna (8th–10th centuries CE). *J. Archaeol. Sci. Rep.* **10**, 483–494 (2016).](https://www.zotero.org/google-docs/?cfMyDP)

[27. Buonincontri, M. P., Pecci, A., Di Pasquale, G., Ricci, P. & Lubritto, C. Multiproxy approach to the study of Medieval food habits in Tuscany (central Italy). *Archaeol. Anthropol. Sci.* **9**, 653–671 (2017).](https://www.zotero.org/google-docs/?cfMyDP)

[28. Maxwell, A. Exploring Variations in Diet and Migration from Late Antiquity to the Early Medieval Period in the Veneto, Italy: A Biochemical Analysis. (University of South Florida, 2019).](https://www.zotero.org/google-docs/?cfMyDP)

[29. Rolandsen, G. L., Arthur, P. & Alexander, M. A tale of two villages: Isotopic insight into diet, economy, cultural diversity and agrarian communities in medieval (11th–15th century CE) Apulia, Southern Italy. *J. Archaeol. Sci. Rep.* **28**, 102009 (2019).](https://www.zotero.org/google-docs/?cfMyDP)

[30. Gismondi, A. *et al.* A multidisciplinary approach for investigating dietary and medicinal habits of the Medieval population of Santa Severa (7th-15th centuries, Rome, Italy). *PLOS ONE* **15**, e0227433 (2020).](https://www.zotero.org/google-docs/?cfMyDP)

[31. Paladin, A. *et al.* Early medieval Italian Alps: reconstructing diet and mobility in the valleys. *Archaeol. Anthropol. Sci.* **12**, 82 (2020).](https://www.zotero.org/google-docs/?cfMyDP)

[32. Riccomi, G. *et al.* Stable isotopic reconstruction of dietary changes across Late Antiquity and the Middle Ages in Tuscany. *J. Archaeol. Sci. Rep.* **33**, 102546 (2020).](https://www.zotero.org/google-docs/?cfMyDP)

[33. Viva, S. *et al.* Project nEU-Med. The Contribution of Isotopic Analysis in the Differential Diagnosis of Anemia, the Case of the Medieval Cemetery of Vetricella (Scarlino, GR) in Tuscany. *Environ. Archaeol.* 1–14 (2021) doi:10.1080/14614103.2020.1867290.](https://www.zotero.org/google-docs/?cfMyDP)

[34. Alexander, M. M., Gerrard, C. M., Gutiérrez, A. & Millard, A. R. Diet, society, and economy in late medieval Spain: Stable isotope evidence from Muslims and Christians from Gandía, Valencia. *Am. J. Phys. Anthropol.* **156**, 263–273 (2015).](https://www.zotero.org/google-docs/?cfMyDP)

[35. Sandias, M. & Müldner, G. Diet and herding strategies in a changing environment: Stable isotope analysis of Bronze Age and Late Antique skeletal remains from Ya’amūn, Jordan. *J. Archaeol. Sci.* **63**, 24–32 (2015).](https://www.zotero.org/google-docs/?cfMyDP)

[36. Ma, Y. *et al.* Isotopic reconstruction of diet at the Vandalic period (ca. 5th–6th centuries AD) Theodosian Wall cemetery at Carthage, Tunisia. *Int. J. Osteoarchaeol.* **31**, 393–405 (2021).](https://www.zotero.org/google-docs/?cfMyDP)

[37. Häberle, S. *et al.* Inter- and intraspecies variability in stable isotope ratio values of archaeological freshwater fish remains from Switzerland (11th–19th centuries AD). *Environ. Archaeol.* **21**, 119–132 (2016).](https://www.zotero.org/google-docs/?cfMyDP)

[38. Mion, L. *et al.* The influence of religious identity and socio-economic status on diet over time, an example from medieval France. *Archaeol. Anthropol. Sci.* **11**, 3309–3327 (2019).](https://www.zotero.org/google-docs/?cfMyDP)

[39. Soncin, S. *et al.* High-resolution dietary reconstruction of victims of the 79 CE Vesuvius eruption at Herculaneum by compound-specific isotope analysis. *Sci. Adv.* (2021) doi:10.1126/sciadv.abg5791.](https://www.zotero.org/google-docs/?cfMyDP)

[40. Fernandes, R., Nadeau, M.-J. & Grootes, P. M. Macronutrient-based model for dietary carbon routing in bone collagen and bioapatite. *Archaeol. Anthropol. Sci.* **4**, 291–301 (2012).](https://www.zotero.org/google-docs/?cfMyDP)

[41. Garnsey, P. & Scheidel, W. *Cities, Peasants and Food in Classical Antiquity: Essays in Social and Economic History*. (Cambridge University Press, 1998).](https://www.zotero.org/google-docs/?cfMyDP)

[42. Sadori, L. & Susanna, F. Hints of economic change during the late Roman Empire period in central Italy: a study of charred plant remains from “La Fontanaccia”, near Rome. *Veg. Hist. Archaeobotany* **14**, 386–393 (2005).](https://www.zotero.org/google-docs/?cfMyDP)

[43. Caracuta, V. & Fiorentino, G. L’analisi archeobotanica nell’insediamento di Faragola (FG): il paesaggio vegetale tra spinte antropiche e caratteristiche ambientali, tra tardoantico e altomedioevo. in *Atti del V Congresso Nazionale di Archeologia Medievale* (eds. Volpe, G. & Favia, P.) 371–377 (All’Insegna del Giglio, 2009).](https://www.zotero.org/google-docs/?cfMyDP)

[44. van der Noort, R. *et al.* Excavations at Le Mura di Santo Stefano, Anguillara Sabazia. *Pap. Br. Sch. Rome* **77**, 159–223 (2009).](https://www.zotero.org/google-docs/?cfMyDP)

[45. Murphy, C., Thompson, G. & Fuller, D. Q. Roman food refuse: urban archaeobotany in Pompeii, Regio VI, Insula 1. *Veg. Hist. Archaeobotany* **22**, 409–419 (2013).](https://www.zotero.org/google-docs/?cfMyDP)

[46. Robinson, M. & Rowan, E. Roman Food Remains in Archaeology and the Contents of a Roman Sewer at Herculaneum. in *A Companion to Food in the Ancient World* (eds. Wilkins, J. & Nadeau, R.) 105–115 (Wiley-Blackwell).](https://www.zotero.org/google-docs/?cfMyDP)

[47. Montanari, M. *Alimentazione e cultura nel Medioevo*. (Laterza, 1988).](https://www.zotero.org/google-docs/?cfMyDP)

[48. King, A. C. Diet in the Roman world: a regional inter-site comparison of the mammal bones. *J. Roman Archaeol.* **12**, 168–202 (1999).](https://www.zotero.org/google-docs/?cfMyDP)

[49. Minniti, C. L’approvvigionamento alimentare a Roma nel Medioevo: analisi dei resti faunistici provenienti dalle aree di scavo della Crypta Balbi e di Santa Cecilia. in *Atti del III Convegno Nazionale di Archeozoologia* (eds. Fiore, I., Malerba, G. & Chilardi, S.) 469–492 (Istituto poligrafico e Zecca dello Stato, 2005).](https://www.zotero.org/google-docs/?cfMyDP)

[50. MacKinnon, M. Consistency and change: zooarchaeological investigation of Late Antique diets and husbandry techniques in the Mediterranean region. *Antiq. Tardive* **27**, 135–148 (2019).](https://www.zotero.org/google-docs/?cfMyDP)

[51. Turrini, A., Leclercq, C. & D’Amicis, A. Patterns of food and nutrient intakes in Italy and their application to the development of food-based dietary guidelines. *Br. J. Nutr.* **81**, S83–S89 (1999).](https://www.zotero.org/google-docs/?cfMyDP)
